# Estimating the effectiveness of self-help groups on the adoption of secondary preventive measures by people living with HIV in Central America, 2012

**DOI:** 10.1186/s12913-020-05235-0

**Published:** 2020-05-24

**Authors:** Mario Salvador Sanchez-Dominguez, Hector Lamadrid-Figueroa, Rene Leyva-Flores, Cesar Infante-Xibille

**Affiliations:** 1 Centro de Investigación en Sistemas de Salud, Instituto Nacional de Salud Pública, Cuernavaca, Mexico; 2 Centro de Investigación en Salud Poblacional, Instituto Nacional de Salud Pública, Avenida Universidad 655, Santa Maria Ahuacatitlan, Cp. 62100 Cuernavaca, Morelos Mexico

**Keywords:** Self-help groups, HIV, Effectiveness, Secondary prevention, Central America

## Abstract

**Background:**

According to UNAIDS, the HIV epidemic has stabilized. This as a result of increased condom use and greater access to coverage for antiretroviral therapy (ART). In Central America, civil society organizations work with self-help groups (SHGs) organized in conjunction with public health services to implement interventions seeking to increase condom use and ART adherence for people living with HIV (PLH).

**Method:**

To analyze the effectiveness of SHGs in Central America aimed on increasing condom use and ART adherence in PLH, We conducted a cross-sectional study using a questionnaire and a random sample of 3024 intervention group and 1166 control group. Based on propensity scoring and one-to-one matching (with replacement), we formed a comparison group to help estimate the effectiveness of the above-mentioned intervention on two outcome variables (condom use and ART adherence). The internal consistency of the results was tested through weighted least squares (WLS) and instrumental variable (IV) regression.

**Results:**

Although bivariate comparisons yielded differences between intervention and control group, we found no evidence that the intervention was effective; nor did we find evidence of a heterogeneous impact among countries after adjusting for propensity scoring and the IV model. The impact observed after performing raw comparisons of the indicators may be attributable to self-selection on the part of PLH rather than to the SHGs strategy. Our results demonstrate that it is imperative to use rigorous intervention evaluation methodology to validate the consistency of results.

**Conclusions:**

The intervention had no impact on the outcome indicators measured. We recommend prioritizing the allocation of economic resources for the implementation of interventions with previously proven effectiveness. We also recommend that future studies explore why the intervention failed to produce the expected impact on condom use and ART adherence.

## Background

According to The Joint United Nations Programme on HIV/AIDS (UNAIDS), the HIV epidemic has stabilized in recent years [1]. This news has been circulating in UNAIDS documents over the past 5 years [1–3]; the main reason adduced for this achievement is increased access to Antiretroviral Therapy (ART) and the adoption of preventive measures - primarily safe sex practices - redounding in fewer new infections [3].

However, increased access to ART will be reflected in improved incidence and mortality indicators only if people living with HIV (PLH) achieve therapeutic adherence. To encourage ART adherence, the World Health Organization (WHO) has recommended the implementation of self-help groups (SHGs) aimed at offering PLH medical follow-up and psychological support [4].

SHGs, often initiated and sponsored by community and civil society organizations (CSOs), generally gather in health units, CSOs installations or other community spaces [5]. Those linked to health units and CSOs are tasked with disseminating information on secondary HIV prevention, strengthening the self-esteem and social skills of PLH, reducing HIV-related stigma and discrimination, promoting the retention of PLH by health services and increasing ART adherence [6, 7].

In a meta-analysis of studies on the effectiveness of SHGs from 1995 to 2014, Bateganya et al. (2015) found a number of benefits including greater PLH retention by health services, improved quality of life and lower rates of HIV-related morbidity, mortality and transmission; nonetheless, the authors concluded that evidence was insufficient to draw decisive conclusions on the subject [5].

In Central America, CSOs have been striving to find a response to HIV for more than a decade. International agencies have provided technical and financial cooperation support to them for implementing strategies to reduce the damage and improve the quality of life of PLH. Deserving special mention is an intervention titled *Developing and strengthening the technical and professional capacities of PLHA (Acronym used in this proposal to designate people living with HIV/AIDS) in order that they may effectively contribute to the HIV/AIDS response, quality of life advancement and poverty reduction in the region, 2008–2012* [8]. Its objectives included increasing condom use and ART adherence among PLH in all seven Central American countries. Henceforth, we will refer to this initiative as “the intervention.”

For the implementation of the intervention in Central America, CSOs have relied on SHGs for PLH, most of them located within or close to health units. Considering that evidence on the effectiveness of HIV/AIDS initiatives based on SHGs is virtually non-existent, particularly in Central America, we decided to evaluate the effectiveness of the intervention. We were interested in ascertaining its potential for increasing secondary preventive measures among PLH [9], specifically condom use at last sexual encounter and ART adherence.

## Methods

This is a secondary data analysis of the cross-sectional survey *Elaboration of the risk profile of people living with HIV in the beneficiary countries of the REDCA+ Regional Program* [10]. The survey was administered to PLH in the seven Central American countries between June and October of 2012. Its aim was to explore the status of this population in terms of socio-demographic characteristics, prevalence of condom use, ART adherence, sexual practices, predominance of HIV-related stigma and discrimination and AIDS-related morbidity. The questionnaire was designed based on the USAID’s Guidelines for Repeated Behavior Surveys in Populations at HIV Risk (see additional files section). Our analysis centered on socio-demographic characteristics, condom use and ART adherence.

For the survey results to be nationally representative, the seven countries were regarded as strata with similar sample sizes. The SHGs were defined as clusters, 103 SHGs were selected from the lists provided by the health authorities of participating countries. The sample size by country was proportional to the number of clusters. It was calculated taking into account that the parameter to be estimated was the proportion of people diagnosed with HIV who had used a condom during their last sexual encounter, which was assumed to be between 40 and 70% regardless of the country. The sampling strategy resulted in a design effect (DEFF) of 1.74.

SHGs were randomly selected and visited; every third individual who arrived at the sessions was interviewed. Based on the records of local health services and references from users PLH who were non-SHGs users were identified and visited at them home where pollsters applied the questionnaires. Data collection in each country lasted 66 days on average, that is, until reaching the required size. The sample collected was 4940 individuals. Unable to make home interviews in Nicaragua, this country was excluded from our study. Final data analyzed pertained to 4190 individuals: 3024 interviewed at 88 SHGs and 1166 at home.

Following data collection, the effectiveness of the intervention was evaluated. Our objective was to assess whether it had contributed to increased condom use and ART adherence among PLH in six Central American countries: El Salvador, Belize, Costa Rica, Guatemala, Honduras and Panama.

The intervention was developed according to a Behavior Change Communication strategy known as *Because I am capable, I act* (the original version was registered by the LLAVES Foundation in Pedro Sula, Honduras, under the following title: *Initiative Based on the Integrated Behavior Development and/or Change Communication Strategy*). The CSOs in charge of implementation at the regional level trained PLH - designated as “focal points” - to offer peer counseling to PLH attending SHGs.

For the purposes of our study, the intervention and control groups was defined as follows:

- Intervention group were PLH users of SHGs who were interviewed at the sites of their sessions, and
- Control group (those who did not form part the intervention) were PLH who did not attend SHGs and were interviewed at home.

Intervention group received medical care and follow-up as well as ART and behavior change counseling under the *Because I am capable, I act* model, with the latter provided permanently by trained personnel. SHGs attendance by participants was estimated at one in-person session per month lasting from 60 to 120 min.

In-person sessions included games designed to promote behavior changes, safe-sex practices and ART adherence. Time was provided at the end of each session for group members to discuss their experiences. For the most part, participants attended sessions following their monthly medical consultation, with the number of attendees ranging from five to ten. During the intervention, which lasted 4 years, PLH were offered medical follow-up and ART at their health units and behavior change counseling in their SHGs.

Because of its potential benefits, the intervention was made available for the entire PLH population attending SHGs, not was a randomly assigned individuals. It is therefore important to consider the presence of a self-selection bias in our research and, hence, the need to isolate factors extraneous to the intervention which might have affected its expected results [11], namely individual characteristics which may have rendered PLH un/likely to self-select themselves for intervention.

Given that the intervention was not randomized, its estimated effects on our outcome variables may have been affected by confounding factors. Nonetheless, in order to minimize bias from observable characteristics, we used propensity score (PS) matching [12–14]. In other words, by setting up a control group (PLH who did not attend SHGs sessions), we were able to contrast our group of participants (intervention) against a subgroup of non-participants (control) exhibiting highly similar covariables or a comparable group.

Insofar as the people in the intervention and control groups pertained to a population of similar individuals as regards eligibility to receive intervention, PS matching proved a valid method for establishing a control group with comparable characteristics. This facilitated our evaluation of the effectiveness of the intervention [12–14]. In order to ensure consistency, we verified that our matching procedure met the required criteria of conditional independence and region of common support [13].

We assumed that the following factors may have influenced the inclusion or likelihood of participating in the intervention: individual characteristics such as demographic profiles [15]; availability and accessibility of medical services [16], depending on the country of residence; a propensity to engage in risky behavior; a history of drug use [17–19]; and resilience factors related to adaption to be living with HIV [20].

The characteristics used in PS estimation were the following:

1. Country of residence: El Salvador, Belize, Costa Rica, Guatemala, Honduras and Panama. We included this information because SHG availability can differ by country;
2. Age: 18–24, 25–44, 45–59 and ≥ 60 years. We grouped participants by generational stage;
3. Schooling: none, literate, 6, 12 or ≥ 16 years of schooling;
4. Sex: male or female;
5. Sexual orientation: heterosexual, bisexual or homosexual. We included these categories because it has been reported that homosexuals and bisexuals exhibit a higher rate of SHGs attendance than do heterosexuals [21];
6. Currently in a stable relationship: yes or no. This characteristic referred to individuals with whom interviewees had sex without payment and maintained an affectionate/constant/regular bond, for example, a partner, boyfriend/girlfriend or spouse, even if not living in the same house. Couple counseling has been recommended by UNAIDS as an effective strategy [1] which may correlate positively with SHGs use;
7. Average monthly income in US dollars: lack of money has been reported as an obstacle to SHGs use [15, 22]. As 13.6% (*n* = 572) of interviewees did not disclose their monthly income, we constructed a zero-inflated Poisson regression model to estimate the missing data on the basis of sex, age, schooling, type of employment and country of residence. With the values obtained we were able to estimate and assign monthly income figures for 86% of the missing values. A 0.65 correlation was obtained between the values predicted by the model and those reported;
8. Family support for dealing with HIV-related disease: yes or no. This item was considered an indicator for family and social support which, in turn, has been reported as a promoter of health-care use [23, 24];
9. Having economic dependents: yes or no. We included this item as an indicator for family structure [23, 24], assuming that PLH who were responsible for economic dependents were more likely to take better care of their health and participate in SHGs;
10. Prior diagnosis of tuberculosis: yes or no. It has been documented that PLH attending SHGs are referred by health personnel working with HIV-related diseases [25];
11. Having received counseling when tested for HIV: yes or no. In the event of a positive result, laboratory staff are required to refer individuals to health services and SHGs [5, 22];
12. History of drug use: yes or no. Use of drugs has been associated with reduced SHGs attendance [1]; and
13. Future Time Perspective (FTP): with values ranging from zero to ten, the FTP scale was used to estimate the resilience of PLH in confronting their condition. FTP is considered a protective factor that promotes resilience, especially among the most vulnerable groups [26]. A negative correlation has been shown to exist between FTP and high-risk sexual practices [27]. People with a highly developed FTP are expected to make better present decisions that will allow them to achieve their future plans. FTP was measured using the Consideration of Future Consequences Scale proposed by Strathman et al. [28] and validated in 2003 by Petrocelli [29].

The Average Treatment Effect on the Treated (ATT) [12] was estimated as a measure of the impact of intervention; that is, the average effect of the intervention solely among intervention group as this was the only part of the sample selected randomly. The ATT was estimated using two indicators: (1) use of a condom at last sexual encounter and (2) ART suspension by own admission, as a means of estimating therapeutic adherence in cases where therapy was not suspended. The two outcome variables were measured through the following questions:

1. Condom use: Section 11 of the questionnaire inquired about the last sexual encounter. Participants were asked to recall their last sexual encounter and the type of partner involved (stable, client, occasional, commercial or stranger). They were then asked, “Did you use a condom the last time you had sexual relations?”
2. Adherence to ART: Section 05 of the questionnaire explored whether participants were currently attending ART, and how long ago they had begun (71% had been attending for less than 5 years). They were then asked if they had suspended ART: “Since you began attending ART, have you ever suspended treatment by your own choice? That is, by your own will and not for reasons of medical prescription?”

The robustness and consistency of the ATT results were tested using four matched algorithms: second nearest neighbor (without specifying caliper value), Kernel (epanechnikov type)*,* one-to-one match (one nearest neighbor) and Weighted Least Squares (WLS) regression. The weights were estimated as follows: value 1 for those who received the intervention and for those who are in the control group the assigned value was 1 / (1-pscore). We used other models to analyze the countries separately (one at a time) in order to identify any heterogeneous effects of the intervention on the two outcome variables.

With a view to reducing the bias generated by omission of explanatory variables, we tested the consistency of the results through a two-stage WLS method incorporating an instrumental variable (IV) which was tested for overidentification, endogeneity and weakness [30].

In order to use this variable as an instrument in IV modeling, we performed a geospatial analysis considering the distance between the places of residence of the PLH and the SHGs as a variable which may have influenced SHGs attendance. The IV employed was the squared Euclidean (straight-line) distance in kilometers between the geographic location of the PLH (locality) and the geographic location of the SHGs [31]. We used the Google Earth Pro 7.7.1 Program to identify the geographic location and georeferenciation of the localities in relation to the SHGs. We also used Qgis 2.18 software to estimate the distances: 578 localities and 89 SHGs were georeferenced, resulting in an estimated 864 distances of interest for the study. For impact estimates, we used the Stata v.13.0 statistical package.

## Results

### Propensity-score (PS) estimation

**Fig. 1:**
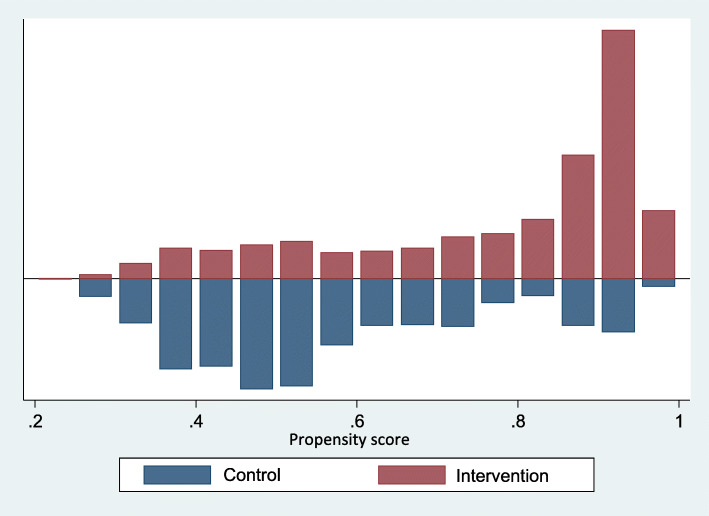
Histogram of the propensity score (region of common support) estimated for the non/participant matching process

**Fig. 2:**
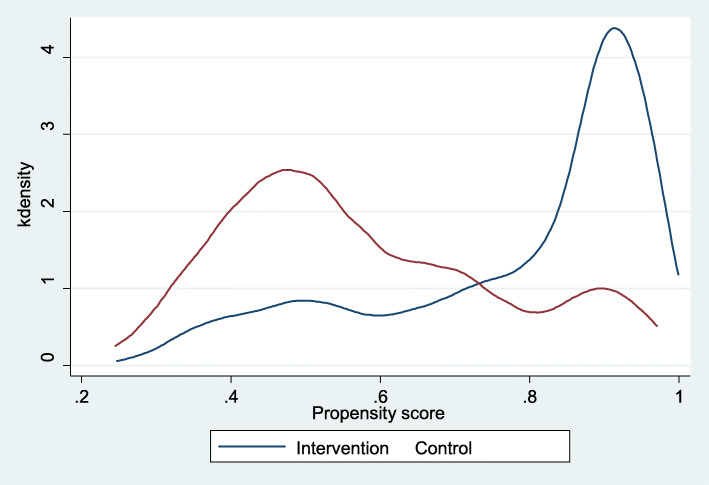
Distribution of observable characteristics in the non/participant groups prior to matching

**Fig. 3:**
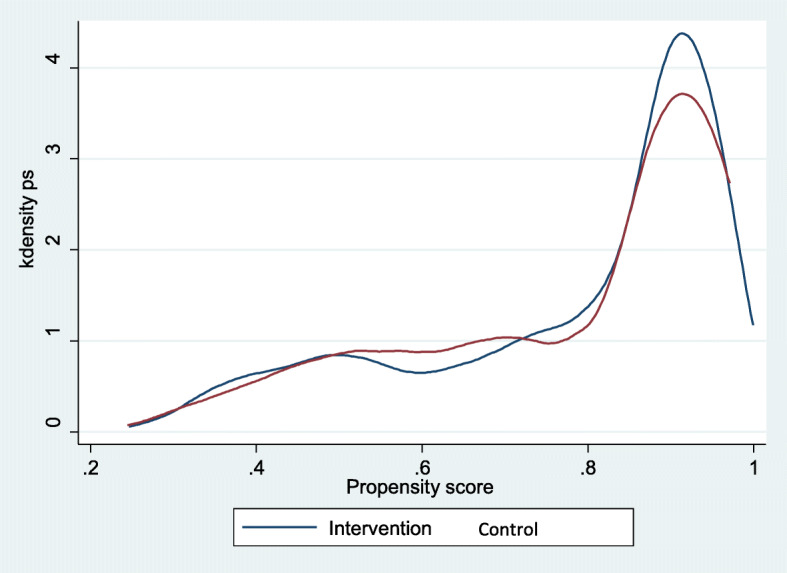
Distribution of observable characteristics in the non/participant groups after matching

We estimated the PS of an initial sample of 4190 observations by adjusting a probit model: 1166 observations pertained to control group and 3024 to intervention group. Based on propensity scoring and one-to-one matching (with replacement), we formed a comparison group. As a result, we identified 1032 PLH in control group and 2629 PLH in intervention group on the region of common support, representing an adequate sample size for both groups. Figure 1 shows the histogram of the estimated PS including the region of common support, while Figs. 2 and 3 show sample distribution before and after matching.

We analyzed PS and matching using the Kernel method to explore the reduction of the differences and the bias of the variables following matching; the general model yielded a bias reduction from 15.6 to 6.2% (*p* < 0.001). Table 1 details the information for each variable.

**Table 1:** Bias reduction tests before and after matching based on the Kernel algorithm

Variable		Intervention	Control	%bias	%bias reduction	t test	*P* value
**Sex (Male)**	Without matching	0.548	0.510	7.7	–	1.97	0.048
	**Kernel**	0.548	0.561	− 2.5	67.0	− 0.89	0.372
**Age (years)**							
Youth (18–24)	Without matching	0.122	0.092	10.0	–	2.48	0.013
	**Kernel**	0.122	0.126	−1.1	89.4	−0.35	0.729
Young adult (25–44)	Without matching	0.623	0.627	−0.9	–	−0.23	0.819
	**Kernel**	0.623	0.626	−0.6	33.9	−0.21	0.836
Mature adult (45–59)	Without matching	0.209	0.243	−8.3	–	−2.15	0.031
	**Kernel**	0.209	0.215	−1.5	82.3	−0.53	0.600
Older adult (≥60)	Without matching	0.046	0.038	4.1	–	1.03	0.302
	**Kernel**	0.046	0.034	6.1	−48.4	2.19	0.029
**Schooling**							
None	Without matching	0.115	0.066	17.2	–	4.17	0.000
	**Kernel**	0.115	0.097	6.4	62.5	2.09	0.036
Literate	Without matching	0.029	0.032	−2.2	–	−0.56	0.573
	**Kernel**	0.029	0.038	−5.2	−140.8	−1.76	0.079
Completed elementary school	Without matching	0.367	0.449	−16.6	–	−4.28	0.000
	**Kernel**	0.367	0.357	2.0	87.9	0.71	0.475
Completed middle school	Without matching	0.383	0.376	1.4	–	0.35	0.724
	**Kernel**	0.383	0.384	−0.2	87.8	−0.06	0.953
University	Without matching	0.106	0.077	10.2	–	2.52	0.012
	**Kernel**	0.106	0.125	−6.4	37.3	−2.01	0.044
Average monthly income	Without matching	205.760	182.180	10.4	–	2.61	0.009
	**Kernel**	205.760	216.680	−4.8	53.7	−1.73	0.083
**Yes, economic dependents**	Without matching	0.634	0.593	8.4	–	2.16	0.031
	**Kernel**	0.634	0.597	7.6	10.0	2.65	0.008
**Sexual orientation**							
Heterosexual	Without matching	0.789	0.749	9.5	–	2.47	0.014
	**Kernel**	0.789	0.706	19.6	−106.2	6.68	0.000
Bisexual	Without matching	0.071	0.066	1.9	–	0.49	0.621
	**Kernel**	0.071	0.088	−7.0	− 258.3	−2.27	0.023
Homosexual	Without matching	14,029.000	0.185	−12.2	–	−3.21	0.001
	**Kernel**	0.140	0.205	−17.6	−44.6	−6.04	0.000
**Yes, received counseling when tested for HIV.**	Without matching	0.851	0.826	6.7	–	1.75	0.080
	**Kernel**	0.851	0.793	15.6	−130.7	5.25	0.000
**Yes, has suffered from tuberculosis.**	Without matching	0.142	0.094	14.9	–	3.66	0.000
	**Kernel**	0.142	0.137	1.4	90.6	0.45	0.650
**Yes, receives family support.**	Without matching	0.744	0.769	−5.8	–	−1.48	0.139
	**Kernel**	0.744	0.751	−1.6	72.2	−0.56	0.576
**Yes, has used drugs.**	Without matching	0.109	0.179	−19.9	–	−5.34	0.000
	**Kernel**	0.109	0.174	−18.5	6.8	−6.51	0.000
**Yes, is currently in a stable relationship.**	Without matching	0.452	0.432	3.9	–	1.01	0.312
	**Kernel**	0.452	0.438	2.8	29.1	0.98	0.328
**Future time perspective**	Without matching	7.310	7.285	1.8	–	0.46	0.647
	**Kernel**	7.310	7.180	9.1	− 410.0	3.12	0.002
**Country**							
El Salvador	Without matching	0.119	0.334	−52.9	–	−14.84	0.000
	**Kernel**	0.119	0.110	2.4	95.4	1.08	0.281
Belize	Without matching	0.102	0.021	34.0	–	7.67	0.000
	**Kernel**	0.102	0.079	9.8	71.1	2.85	0.004
Costa Rica	Without matching	0.087	0.287	−53.1	–	−15.25	0.000
	**Kernel**	0.087	0.095	−2.2	95.9	−1.00	0.320
Guatemala	Without matching	0.253	0.060	55.0	–	12.60	0.000
	**Kernel**	0.253	0.237	4.5	91.7	1.30	0.195
Honduras	Without matching	0.223	0.241	−4.3	–	−1.11	0.267
	**Kernel**	0.223	0.230	−1.6	61.7	−0.58	0.561
Panama	Without matching	0.216	0.057	47.6	–	10.95	0.000
	**Kernel**	0.216	0.250	−10.2	78.7	−2.81	0.005

Source: prepared by the author based on data from the following project: *Elaboration of the risk profile of people living with HIV in the beneficiary countries of the REDCA+ Regional Program*

Information was obtained running the Stata 13.1 pstest command. Data were matched using the Kernel method after estimating the propensity score through a probit model. Matching took into account the region of common support and the balance of the variables presented in this table. Mean bias dropped from 15.6 to 6.2

### Socio-demographic characteristics, sexual behavior and health

The sample analyzed was composed largely of young adults (25–44 years old) with 6–12 years of schooling and an average monthly income of 193 US dollars. In the majority of cases, they were responsible for economic dependents. Six out of ten mentioned having no stable partner at the moment of the interview.

Eight out of ten interviewees reported having received counseling when they learned they were seropositive, and only 12% of interviewees mentioned having a history of pulmonary tuberculosis. The majority related that they could count on their families for care in the event of hospitalization. Table 2 details population characteristics before and after sample matching.

**Table 2:** General characteristics of the analyzed sample

		Intervention-differentiated population					
Variables*	General total *n* = 4190	Not matched		Difference test P value	Matched		Difference test P value
		Control *n* = 1166	Intervention *n* = 3024		Control *n* = 1032	Intervention *N* = 2629	
**Sex**							
Female	1894	547 [47]	1347 [45]	0.155	497 [48]	1193 [45]	0.129
**Age groups**							
Youth (18–24 years)	484	111 [10]	373 [12]	0.013	94 [9]	322 [12]	0.014
Young adult (25–44 years)	2601	735 [63]	1866 [62]		649 [63]	1629 [62]	
Mature adult (45–59 years)	899	272 [23]	627 [21]		247 [24]	550 [21]	
Older adult (≥60 years)	206	48 [4]	158 [5]		42 [4]	128 [5]	
**Schooling**							
None	402	72 [6]	330 [11]	< 0.001	66 [6]	301 [11]	< 0.001
Literate	142	41 [4]	101 [3]		36 [3]	86 [3]	
6 years	1540	487 [43]	1053 [32]		446 [43]	958 [36]	
12 years	1611	435 [38]	1176 [40]		397 [38]	1011 [33]	
≥ 16 years	417	105 [9]	312 [11]		87 [8]	273 [10]	
**Average monthly income**	197	177 [219]	204[234]	< 0.001	172 [211]	200[233]	< 0.001
**Economic dependents**							
Yes	2567	665 [57]	1902 [63]	< 0.001	584 [57]	1635 [62]	0.002
**Sexual orientation**							
Heterosexual	3126	837 [75]	2289 [79]	< 0.001	779 [75]	2083 [79]	0.017
Bisexual	289	84 [7]	205 [7]		74 [7]	187 [7]	
Homosexual	611	200 [18]	411 [14]		179 [17]	359 [14]	
**Received counseling when tested for HIV**							
Yes	3533	973 [83]	2560 [85]	0.335	866 [84]	2245 [85]	0.260
**History of pulmonary tuberculosis**							
Yes	500	100 [9]	400 [13]	< 0.001	90 [9]	359 [14]	< 0.001
**Family support for dealing with disease**							
Yes	3068	886 [79]	2182 [75]	0.012	805 [78]	1960 [75]	0.029
**History of drug use**							
Yes	490	174 [15]	316 [11]	< 0.001	168 [16]	277 [11]	< 0.001
**Currently in a stable relationship**							
Yes	1703	437 [39]	1266 [43]	0.007	406 [39]	1137 [43]	0.031
**Future Time Perspective (FTP)**	7.27	7.20 [1.5]	7.30 [1.4]	0.076	7.31 [1.4]	7.34 [1.4]	0.594
**Country of residence**							
El Salvador	797	422 [36]	375 [12]	< 0.001	389 [38]	339 [13]	< 0.001
Belize	398	51 [5]	347 [11]		32 [3]	292 [11]	
Costa Rica	624	330 [28]	294 [10]		276 [27]	234 [9]	
Guatemala	792	70 [6]	722 [24]		59 [6]	641 [24]	
Honduras	799	225 [19]	574 [19]		220 [21]	557 [21]	
Panama	780	68 [6]	712 [24]		56 [5]	566 [22]	
***Outcome variables***					–	–	–
**Condom use**					–	–	–
Yes	2745	693[60]	2052 [67]	< 0.001	–	–	–
**ART Adherence**					–	–	–
Yes	2878	829 [71]	2049[68]	< 0.001	–	–	–

*Estimates for the variables of interest were performed at sample level. Proportions are presented in brackets. In the case of income and FTP, standard errors are presented in brackets. Source: prepared by the author based on data from the following project: *Elaboration of the risk profile of people living with HIV in the beneficiary countries of the REDCA+ Regional Program*

### Impact of intervention on condom use at last sex

**Fig. 4:**
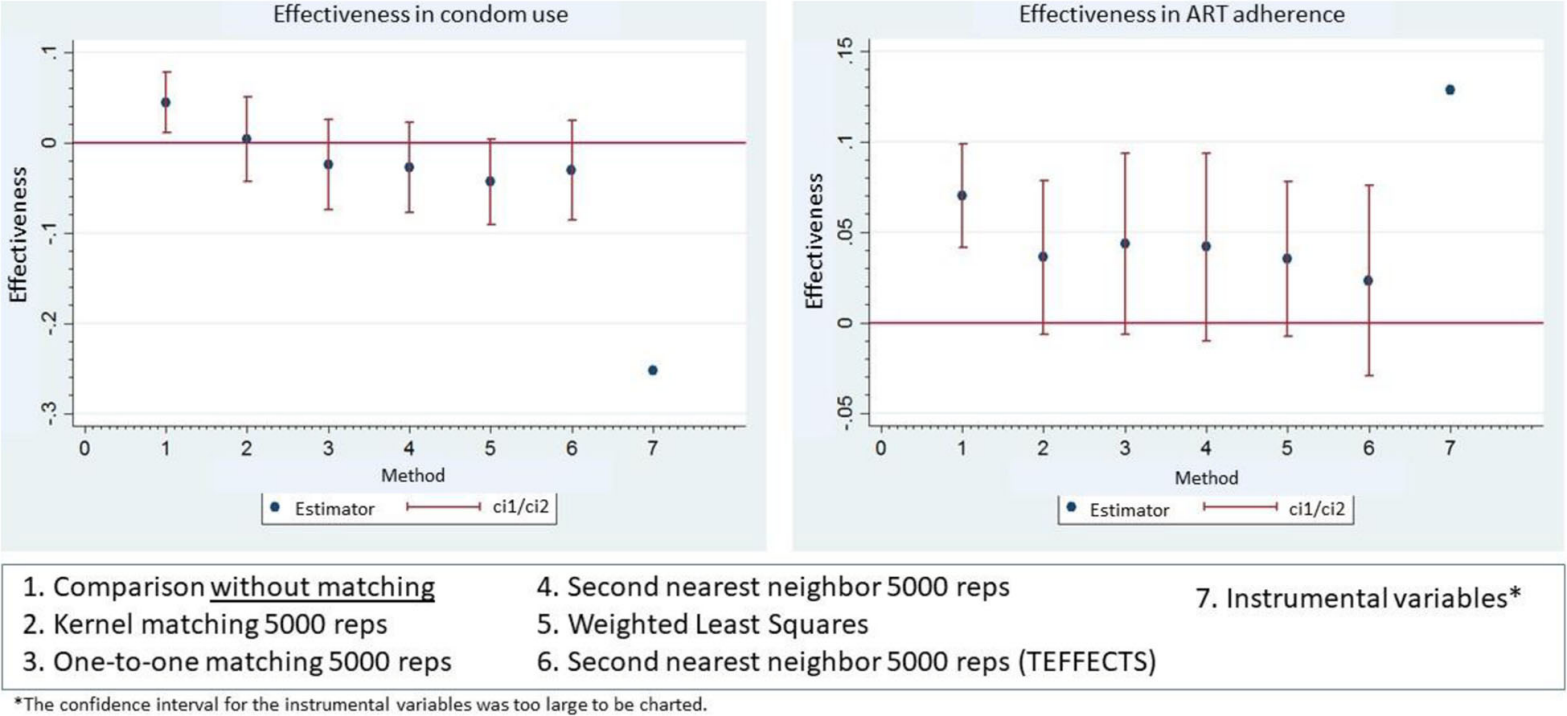
Effectiveness of the intervention according to outcome indicators

In analyzing the average impact of the intervention on condom use at last sex among those being treated (ATT), no significant impact emerged from any of the four methods. Kernel matching yielded an estimator of 4.4 positive percentage points (95% CI -0.043/0.050) and WLS one of 4.3 negative percentage points (95% CI -0.091/0.004). The other methods generated estimators in the range of − 0.006 to 0.073, as shown in Fig. 4. No heterogeneous effects were observed as a result of the intervention in the sampled countries (Table 3).

**Table 3:** Average effect of treatment for those treated (ATT) on the outcomes measured by country

	Condom use		Adherence	
Country	Estimator	*P* Value	Estimator	*P* Value
El Salvador	−0.061	0.116	−.0030	0.368
Belice	0.293	0.132	−0.181	0.472
Costa Rica	0.024	0.607	−0.007	0.808
Guatemala	− 0.069	0.239	0.0312	0.556
Honduras	−.0028	0.366	0.013	0.624
Panama	0.068	0.299	0.159	0.155
Regional (all countrys)	0.004	0.877	0.036	0.096

Source: prepared by the author based on data from the following project: *Elaboration of the risk profile of people living with HIV in the beneficiary countries of the REDCA+ Regional Program*; Kernel matching (500 reps)

The IV method generated the most imprecise estimator with a 95% CI of 1.296 to 0.792. This method yielded a significant squared Euclidean distance association between the geographic location of the place of residence of the individual and the geographic location of the SHG attended, confirming that PLH used nearby SHGs less than distant ones. This made it possible to verify the validity of the instrument employed (Table 4).

**Table 4:** Ordinary Least Squares model weighted in two stages with instrumental variables incorporated for condom use

Variables	Categories		1st stage: Intervention vs. control			2nd stage: Condom use		
			**Coeff**	**CI**		**Coeff**	**CI**	
**Country**	El Salvador		1.000			1.000		
	Belice		0.453*	0.397	0.508	0.343	−0.131	0.818
	Costa Rica		− 0.042	− 0.111	0.025	− 0.079	− 0.162	0.002
	Guatemala		0.426*	0.374	0.477	0.144	−0.293	0.581
	Honduras		0.230*	0.177	0.283	0.184	−0.063	0.433
	Panama		0.432*	0.380	0.483	0.224	−0.227	0.675
**Age (years)**	16–24		1.000			1.000		
	25–44		−0.033	−0.077	0.010	0.062	−0.001	0.127
	45–59		−0.046	−0.099	0.006	0.023	−0.054	0.100
	≥60		− 0.050	−0.132	0.031	−0.037	− 0.147	0.072
**Schooling**	None		1.00			1.000		
	Literate		−0.015	−0.107	0.076	0.042	−0.080	0.164
	6 years		−0.066*	−0.110	− 0.022	0.016	− 0.073	0.105
	12 years		− 0.116*	−0.164	− 0.068	0.036	− 0.095	0.167
	≥16 years		−0.057	− 0.119	0.004	0.044	−0.052	0.141
**Sex**	Female		1.000			1.000		
	Male		0.035*	0.000	0.070	0.122*	0.071	0.172
**Sexual orientation**	Heterosexual		1.000			1.00		
	Bisexual		0.029	0.029	−0.035	0.054	− 0.020	0.129
	Homosexual		0.007	0.007	−0.040	0.099*	0.042	0.157
**Currently in a stable relationship**	No		1.000			1.000		
	Yes		0.017	−0.013	0.048	0.201*	0.164	0.238
**Average** **Monthly income**	0–2581 USD		−0.000	− 0.000	0.000	0.000	−5.480	0.000
**Family support**	No		1.000			1.000		
	Yes		0.037*	0.002	0.071	0.022	−0.031	0.076
**Economic dependents**	No		1.000			1.000		
	Yes		−0.009	− 0.042	0.024	0.021	−0.016	0.060
**History of pulmonary tuberculosis**	No		1.000			1.000		
	Yes		0.029	−0.011	0.070	− 0.056*	− 0.111	− 0.001
**Received counseling when tested for HIV**	No		1.000			1.000		
	Yes		−0.023	−0.065	0.019	0.024	− 0.029	0.078
**History of drug use**	No		1.000			1.000		
	Yes		−0.037	−0.092	0.016	0.045	−0.019	0.110
**Future time perspective (scale)**		1	0.058	−0.054	0.172	0.662*	0.403	0.922
		2	−0.235*	− 0.463	− 0.006	0.588*	0.197	0.980
		3	−0.269*	−0.531	− 0.006	0.472*	0.065	0.878
		4	−0.252*	−0.418	− 0.086	0.746*	0.431	1.061
		5	−0.260*	−0.376	− 0.144	0.647*	0.351	0.943
		6	−0.215*	−0.307	− 0.122	0.626*	0.379	0.873
		7	−0.182*	−0.270	− 0.094	0.718*	0.504	0.933
		8	−0.152*	−0.243	− 0.062	0.797*	0.611	0.983
		9	−0.265*	−0.354	− 0.175	0.845*	0.554	1.136
		10	−0.133*	−0.237	− 0.028	0.700*	0.489	0.910
**Distance to SHG**	Km (Euclidean)		−0.000*	−0.001	− 0.000	–	–	–
**Distance to SHG - squared Euclidean**	Km (Euclidean)		4.170*	1.140	7.210	–	–	–
**Participation**	Control		–	–	–			
	Intervention		–	–	–	−0.252	−1.296	0.791

Source: prepared by the author based on data from the following project: *Elaboration of the risk profile of people living with HIV in the beneficiary countries of the REDCA+ Regional Program;* *p < 0.05; overidentification test: *p* = 0.861; endogeneity test: *p* = 0.265; weakness test: *p* = 0.024 (F = 3.71)

### Impact of the intervention on ART adherence

Voluntary suspension of ART, which was employed as both a proxy for measuring ART adherence and the second outcome variable, did not yield statistically significant estimators. Kernel matching produced an estimated impact of 3.6 percentage points (95% CI-0.006/0.079), while the WLS yielded an estimated impact of 3.5 percentage points (95% CI-0.008/0.078). The other methods produced results ranging from − 0.007 to 0.072 (Fig. 4). No heterogeneous effects from the intervention were identified in the sampled countries (Table 3).

When modeling the outcome indicator using an IV, we also found a significant association of the instrument employed. The values resulting from the tests performed on the IV model for adherence (squared Euclidean distance) are shown in Table 5. The consistency of the parameters for both outcome indicators was tested by performing 5000 bootstrap replications in ATT estimation (Table 6).

**Table 5:** Ordinary Least Squares model weighted in two stages with instrumental variables incorporated for therapeutic adherence

Variables	Categories		1st stage: Intervention vs. control			2nd stage: adherence		
			**Coeff**	**CI**		**Coeff**	**CI**	
**Country**	El Salvador		1.000			1.000		
	Belize		0.467*	0.410	0.524	−0.085	−0.439	0.269
	Costa Rica		−0.046	−0.115	0.022	−0.059	− 0.121	0.002
	Guatemala		0.435*	0.382	0.487	−0.035	− 0.356	0.286
	Honduras		0.249*	0.195	0.303	−0.072	− 0.268	0.123
	Panama		0.422*	0.370	0.475	0.258	−0.062	0.580
**Age (years)**	16–24		1.000			1.000		
	25–44		−0.055*	−0.097	− 0.012	0.026	− 0.033	0.086
	45–59		−0.072*	−0.123	− 0.021	0.019	− 0.052	0.0918
	≥60		−0.066	−0.143	0.009	0.004	−0.080	0.089
**Schooling**	None		1.000			1.000		
	Literate		−0.025	−0.126	0.074	0.037	−0.039	0.115
	6 years		−0.061*	−0.106	− 0.017	0.044	− 0.014	0.104
	12 years		−0.104*	−0.153	− 0.056	0.070	− 0.012	0.153
	≥16 years		−0.044	−0.107	0.017	0.022	−0.046	0.090
**Sex**	Female		1.000			1.000		
	Male		0.028	−0.005	0.062	−0.017	−0.054	0.020
**Sexual orientation**	Heterosexual		1.000			1.00		
	Bisexual		0.056	−0.005	0.119	−0.013	−0.090	0.064
	Homosexual		0.013	−0.032	0.059	−0.015	−0.067	0.036
**Currently in a stable relationship**	No		1.000			1.000		
	Yes		0.019	−0.012	0.050	−0.020	−0.050	0.009
**Average** **Monthly income**	0–2581 USD		−8.720	−0.000	0.000	−8.870	−0.000	0.000
**Family support**	No		1.000			1.000		
	Yes		0.036*	0.001	0.071	−0.032	−0.072	0.007
**Economic dependents**	No		1.000			1.000		
	Yes		−0.006	−0.039	0.027	0.035*	0.005	0.066
**History of pulmonary tuberculosis**	No		1.000			1.000		
	Yes		0.015	−0.024	0.056	0.081*	0.038	0.125
**Received counseling when tested for HIV**	No		1.000			1.000		
	Yes		−0.005	−0.049	0.039	−0.057*	− 0.099	−0.015
**History of drug use**	No		1.000			1.000		
	Yes		−0.034	−0.090	0.021	0.136*	0.069	0.202
**Future time perspective (scale)**								
		1	0.198*	0.099	0.297	−0.166	−0.369	0.036
		2	0.052	−0.149	0.254	−0.162	−0.343	0.017
		3	−0.053	−0.256	0.148	−0.040	− 0.239	0.159
		4	−0.063	−0.213	0.086	−0.021	− 0.193	0.150
		5	−0.116*	−0.224	− 0.007	−0.103	− 0.257	0.050
		6	−0.057	−0.132	0.017	−0.048	− 0.175	0.077
		7	−0.033	−0.106	0.038	−0.083	− 0.201	0.034
		8	−0.016	−0.092	0.059	−0.085	− 0.203	0.032
		9	−0.113*	−0.193	− 0.033	−0.080	− 0.225	0.063
		10	0.198*	0.099	0.297	−0.166	−0.369	0.036
**Distance to SHG**	Km (Euclidean)		−0.001*	−0.001	− 0.000	–	–	–
**Distance to SHG - squared Euclidean**	Km (Euclidean)		4.560*	1.410	7.720	–	–	–
**Participation**	Control		–	–	–	1.000		
	Intervention		–	–	–	0.128	−0.629	0.885

Source: prepared by the author based on data from the following project: *Elaboration of the risk profile of people living with HIV in the beneficiary countries of the REDCA+ Regional Program;* *p < 0.05; overidentification test: *p* = 0.282; endogeneity test: *p* = 0.756; weakness test: *p* = 0.012 (F = 3.37)

**Table 6:** Average effect of treatment for those treated (ATT)

Indicator	Method	Sample Size	Estimator	*P* value	Standard Error	Confidence Interval	
Condom use at last sex	**Without matching**	**3341**	**0.044**	**0.005**	**0.017**	**0.011**	**0.078**
	Kernel (5000 reps*)	3341	0.004	0.877	0.024	−0.043	0.050
	One-to-one (5000 reps)	3341	−0.025	0.336	0.026	−0.075	0.025
	2nd nearest neighbor (5000 reps)	3341	−0.028	0.274	0.025	−0.078	0.022
	Weighted OLSs¥**	3340	−0.043	0.073	0.024	−0.091	0.004
	2nd nearest neighbor (TEFFECTS***)	3341	−0.030	0.279	0.028	−0.085	0.025
	Instrumental variables ¥	3326	−0.252	0.636	0.533	−1.296	0.792
	Kernel 5000 reps (ATTK***)	3660	0.006	0.412	0.026	−0.045	0.057
Adherence to antiretroviral therapy (ART)	**Without matching**	**3128**	**0.070**	**0.000**	**0.015**	**0.041**	**0.099**
	Kernel (5000 reps)	3128	0.036	0.096	0.022	−0.006	0.079
	One-to-one (5000 reps)	3128	0.044	0.086	0.026	−0.006	0.094
	2nd nearest neighbor (5000 reps)	3128	0.042	0.113	0.026	−0.010	0.093
	WLS ¥	3127	0.035	0.107	0.022	−0.008	0.078
	2nd nearest neighbor (TEFFECTS)	3128	0.023	0.383	0.027	−0.029	0.076
	Instrumental variables ¥	3119	0.128	0.740	0.387	−0.629	0.886
	Kernel 5000reps (ATTK)	3128	0.023	0.175	0.025	−0.026	0.072

Source: prepared by the author based on data from the project, *Elaboration of the risk profile of people living with HIV in the beneficiary countries of the REDCA+ Regional Program*

*reps bootstrap replications

**¥Estimates were based on sample weights

***TEFECTS Stata command

****ATTK effect of treatment for those treated by Kernel matching

Because temporal measurement of adherence is imprecise, we performed a second analysis. In this case, we used solely the subsample of PLH who had attended ART for less than 5 years (2027), the approximate duration of the intervention. No statistical difference was observed in the estimates for intervention vs. control group. The ATTK obtained through Kernel matching was 0.024 with a *p* value of 0.434, while that obtained through nearest neighbor matching was 0.003 with a p value of 0.925.

## Discussion

Our results indicated that the intervention under study did not exert an impact on the outcome indicators measured. This finding contradicts the results that would have ensued from a simple comparative analysis of indicators; such an analysis would have yielded positive impacts of at least five percentage points on condom use and seven percentage points on ART adherence.

Because SHGs participation was voluntary and therefore not random, the results above may be explained more in terms of an association with individual characteristics such as FTP and self-selection for intervention than in terms of a direct impact from the strategy under evaluation. In other words, it is possible that those who decided to participate in the intervention had greater interest in caring for their health and were therefore more inclined to use condoms and adhere to ART than those who refrained from participating.

Our results differ from those presented in a study published by Lung Vu et al. in 2015 [34]. This study found that interventions based on the Behavior Change Communication (BCC) model were effective in increasing condom use up to 2.4 times. This degree of effectiveness may be a result of having employed only one matching methodology and not considering the importance of the specific PLH involved as well as other variables including educational and income levels when forming the control group. It is thus possible that self-selection bias was overlooked by these authors, something we took into account in formulating the methodological approach for our study.

The main limitation of our study was that no baseline measurement was taken prior to the intervention, with the only data available dating from a one time-point measures taken in 2012, 4 years after initiating the intervention. It is for this reason that we used several complementary methods for estimating indicators as a way of reducing biases related to the lack of an experimental design, the absence of a baseline measurement and the possibility of self-selection. In this respect, we controlled extensively for observable characteristics that, in theory, could have influenced self-selection. We also used methodology appropriate for cross-sectional studies, achieving a substantial reduction by forming a control group. In all cases, we obtained consistent estimators but observed no significant impact from the intervention under any of the methods utilized. Neither did we find any heterogeneous effects upon analyzing the countries separately.

Although the variable employed as an instrument – Euclidean distance – was weak, it proved significant in both cases. This means that PLH do not always use the nearest health services but are willing to travel a bit farther to receive services [31]. Other studies have yielded similar results, explaining them by reference to the desire of PLH to preserve their anonymity, obtain specialized services, guarantee the availability of medications or receive care in top-level facilities [32, 35, 36]. According to Cook et al. [37], more than half of the population do not use the nearest health facility, and people at higher socioeconomic levels tend to travel greater distances to seek care.

Civil society organizations (CSOs) play a predominant role in the response to HIV. It is important to consider that the intervention analyzed had objectives beyond those evaluated in this study, namely the participation of PLH in the regional response to HIV/AIDS, social mobilization and the enforcement of the rights and responsibilities of PLH (objectives extracted from the funding proposal document). It would be useful to evaluate these objectives in order to determine which ones have achieved positive results and support their continuity with scientific evidence.

UNAIDS suggests that reducing the number of new HIV infections by 2020 will require investing at least a quarter of the total HIV budget in prevention, and has stressed the importance of allocating funds only for interventions of proven effectiveness [38]. As a consequence, international financial organizations should support shifting financing to proposals for interventions whose effectiveness has been scientifically proven.

Given potential bias from omitted (not observable) variables, we used an IV model to analyze the possible impact of intervention by controlling for geographic distance. Even if Euclidean distance does not consider environmental factors such as means of communication and transportation, it is nonetheless a valid way of identifying general differences resulting from geographic proximity to health units [31].

It is likely that the outcome variable, therapeutic adherence, represents a measurement error as a result of self-reporting. However, this would not be expected to constitute a differential in the intervention and control groups, as participating was not conditioned on a response to this variable. Moreover, evidence indicates that self-reporting overestimates real adherence by as much as 25% [33]; if a significant impact had been found, appropriate corrections could have been made. Furthermore, we recognize the problems of temporality associated with measuring this variable; for this reason we undertook an additional analysis with a subsample of PLH who had been receiving ART less than 5 years, and still found no significant impact.

Finally, we recognize that lack of a baseline measurement rendered it impossible to determine either the parameter of the effectiveness indicator prior to the intervention or what occurred between 2008 and 2012. As a consequence, we only analyzed the final results observed in 2012.

## Conclusions

Our study results contribute empirical evidence regarding the effectiveness of an HIV/AIDS intervention undertaken in Central America, while highlighting the importance of employing a strict methodology for evaluating the impact of interventions. Little published evidence exists concerning evaluations of interventions of this type in the region.

The intervention under study did not exert an impact on the outcome indicators measured. We suggest evaluating the effectiveness of the interventions already implemented or that international agencies grant financing to implement interventions that have already been proven effective. We also recommend that future studies explore the reasons why the intervention failed to produce the expected impact on condom use and ART adherence.

## Supplementary information

**Additional file 1.** Questionnaire Risk Profile of people living with HIV for the beneficiary countries of the REDCA+ Regional Program.

## Data Availability

The datasets generated and analyzed during this study are not publicly available in order to protect participant anonymity. They consist of questionnaires containing participant information whose publication requires prior authorization. However, they are available from the corresponding author on reasonable request.

## Abbreviations

UNAIDS: United Nations Programme on HIV/AIDS
HIV: Human Immunodeficiency Virus
ART: Antiretroviral Therapy
SHGs: Self-help groups
PLH: People Living with HIV
WLS: Weighted Least Squares
IV: Instrumental Variable
WHO: World Health Organization
CSOs: Community and Civil Society Organizations
HIV/AIDS: Human immunodeficiency virus/Acquired immunodeficiency syndrome
REDCA+: Central American Net of People with HIV
AIDS: Acquired immunodeficiency syndrome
USAID: United States Agency to International Development
DEFF: Design Effect
PS: Propensity Score
FTP: Future Time Perspective
ATT: Average Treatment Effects on the Treated
BCC: Behavior Change Communication
COMISCA: Central America Ministers of Health Council
CI: Confidence Interval
Coeff: Coefficient
