# Supplementary material for: Estimating the effectiveness of self-help groups on the adoption of secondary preventive measures by people living with HIV in Central America, 2012
Source: BMC Health Serv Res. 2020 May 24;20:451. doi: 10.1186/s12913-020-05235-0 (PMC7245741; doi:10.1186/s12913-020-05235-0)
Supplement: Supplementary file 1 — Additional file 1. Questionnaire Risk Profile of people living with HIV for the beneficiary countries of the REDCA+ Regional Program. [file 12913_2020_5235_MOESM1_ESM.pdf]

Risk Profile of people living with HIV for the beneficiary countries of the REDCA+ Regional Program

Country:
BE Belize HO Honduras SA El Salvador
CR Costa Rica NI Nicaragua
GU Guatemala PA Panama

Place where data is collected: 1-( ) Health Centre / Code of Health Centre

2-( ) Home Visit

Interviewer code: ___________________________

DATE:____/____/ __/

Interview time: ___H/___M

Informed consent obtained: 1___Yes, 2___No

The participant enters the study: 1___Yes, 2___No

Condition of the questionnaire: 1__complete, 2__Incomplete

Section 1: Participation in Support groups and perspectives to future plans

We will begin the interview with questions about your participation in support groups. Remember that your answers will be confidential and anonymous.

No.	Questions	Categories	Go to
**P101**	Do you attend a support group for people with HIV?	1- ( ) Yes<br>2- ( ) No……………………………………<br>0- ( ) No response………………………………	→ **P103**
**P102**	What type of group is it?	1- ( ) From the Health Centre<br>2- ( ) From an NGO<br>3- ( ) An independent and spontaneous group<br>0- ( ) No response	
**P103**	In the last year, have you been involved in education on HIV/ Aids or information, such as lectures, workshops?	1- ( ) Yes<br>2- ( ) No<br>0- ( ) No response	
**P104**	Do you think you can contribute something to stop the epidemic of HIV-AIDS?	1- ( ) Yes<br>2- ( ) No……………………………………<br>0- ( ) No response………………………………	→ **P106**
**P105**	How can you contribute to the control of HIV - AIDS?<br><br>**PROBE FOR EACH ACTION AND CHECK THOSE THAT THE PERSON AGREES TO**	- ( ) Using condoms in all my sexual acts<br>- ( ) Taking my medications without interruptions<br>- ( ) Being faithful and having one partner<br>- ( ) Not having sex<br>- ( ) Actively participating with an NGO<br>- ( ) Taking care of my health<br>- ( ) There is nothing I can do<br>- ( ) Other/Specify_________________<br><br>0- ( ) No response	
**P106**	Do you have specific plans for the future?	1- ( ) Yes<br>2- ( ) No<br>0- ( ) No response	
**P107**	Do you think that you can do something positive to change your future?	1- ( ) Yes<br>2- ( ) No<br><br>0- ( ) No response	
**P108**	Do you think that in the future you will be better, worse or the same as now?	1- ( ) Better<br>2- ( ) Worse<br>3- ( ) Same<br><br>4- ( ) Does not know<br>0- ( ) No response	

CONSIDERATION OF FUTURE CONSEQUENCES SECTION

Below we are going to read some statements and ask you to think about how much you agree or disagree with them, to respond consider what you think or would do if you were in similar situations
The scale of answers that I could give are the following:

**1) I totally disagree**<br>**2) I disagree**<br>**3) I couldn't tell**<br>**4) I agree**<br>**5) I totally agree**			
**P109**	When I want to achieve something, I set myself goals and consider the means to achieve them.	1- ( ) I totally disagree<br>2- ( ) I disagree<br>3- ( ) I couldn't tell<br>4- ( ) I agree<br>5- ( ) I totally agree	
**P110**	Preparing for work for the next day and meeting deadlines takes precedence over fun tonight	1- ( ) I totally disagree<br>2- ( ) I disagree<br>3- ( ) I couldn't tell<br>4- ( ) I agree<br>5- ( ) I totally agree	
**P111**	It bothers me a lot to be late for my appointments or commitments.	1- ( ) I totally disagree<br>2- ( ) I disagree<br>3- ( ) I couldn't tell<br>4- ( ) I agree<br>5- ( ) I totally agree	
**P112**	I fulfill on time with the obligations that I have with my friends and bosses.	1- ( ) I totally disagree<br>2- ( ) I disagree<br>3- ( ) I couldn't tell<br>4- ( ) I agree<br>5- ( ) I totally agree	
**P113**	Before making a decision I assess the costs and benefits.	1- ( ) I totally disagree<br>2- ( ) I disagree<br>3- ( ) I couldn't tell<br>4- ( ) I agree<br>5- ( ) I totally agree	
**P114**	I finish my projects on time, progressing steadily and continuously.	1- ( ) I totally disagree<br>2- ( ) I disagree<br>3- ( ) I couldn't tell<br>4- ( ) I agree<br>5- ( ) I totally agree	
**P115**	I make lists of things to do.	1- ( ) I totally disagree<br>2- ( ) I disagree<br>3- ( ) I couldn't tell<br>4- ( ) I agree<br>5- ( ) I totally agree	
**P116**	I am able to resist temptations when I know there is work to be done.	1- ( ) I totally disagree<br>2- ( ) I disagree<br>3- ( ) I couldn't tell<br>4- ( ) I agree<br>5- ( ) I totally agree	
**P116**	I keep working on uninteresting tasks, if they help me get ahead.	1- ( ) I totally disagree<br>2- ( ) I disagree<br>3- ( ) I couldn't tell<br>4- ( ) I agree<br>5- ( ) I totally agree	

SECTION 2 SEXUAL BEHAVIOR

Now I would like to ask you some personal questions about their sex life. Remember that your responses will remain confidential and the questionnaire is anonymous.

No.	Questions	Categories	Go to
P201	Have you had sex that leads to penetration; with penis in vagina, anus or mouth?	1- ( ) Yes<br>2- ( ) No……………………………………<br>0- ( ) No response………………………………	→ Secc. 3
P202	At what age did you have your f irst sexual encounter?	Age in years: _________<br><br>00- ( ) No response	
The following questions are for those that started having sex being minors			
P203	Was your partner, on that f irst sexual act an adult?	1- ( ) Yes<br>2- ( ) No<br>3- ( ) Does not remember<br>0- ( ) No response	
P204	What was your relationship with the partner with whom you had sexual intercourse for the f irst time?	1- ( ) Boyfriend/ Girlfriend<br>2- ( ) Friend / acquaintance<br>3- ( ) Unknown person<br>4- ( ) Person who paid<br>5- ( ) Spouse or husband / wife<br>6- ( ) Family member but not spouse<br>7- ( ) Other /Specify _<br>0- ( ) No response	
P205	In terms of sexual activity...?<br><br>**READ THE ALTERNATIVES**	1-( ) You only have sex with men<br>2-( ) You have sex with men and occasionally with women<br>3-( ) You have sex with men and women<br>4-( ) You have sex with women and occasionally with men<br>5-( ) You only have sex with women<br>0- ( ) No response	
**SECTION FOR WOMEN BEGINS (P206 a P213)**<br><br>**IF YOU ARE INTERVIEWING A MAN GO TO → SECTION 3**			
P206	Have you been pregnant after knowing your diagnosis of HIV?	1- ( ) Yes<br>2- ( ) No……………………………………<br>0- ( ) No response………………………………	→ P212
P207	How many pregnancies have you had after being diagnosed HIV +?	Number of pregnancies : _________<br><br>0- ( ) No response	
P208	Did you plan that or those pregnancies?	1- ( ) All the time<br>2- ( ) Most of the times<br>3- ( ) Few times<br>4- ( ) Never<br>0- ( ) No response	

No.	Questions	Categories	Go to
**P209**	In your last pregnancy did you receive information about the risk of HIV transmission f rom mother to child?	1- ( ) Yes<br>2- ( ) No<br><br>0- ( ) No response	
**P210**	How many of the children you have had since your diagnosis are HIV +?	Number of children : _________<br><br>0- ( ) No response	
**P211**	How many Pop Smears has been made in the last twelve months?	1- ( ) 0<br>2- ( ) 1<br>3- ( ) 2<br>4- ( ) More than 2<br>0- ( ) No response	
**P212**	Are you currently using any method of family planning?	1- ( ) Yes<br>2- ( ) No...<br>0- ( ) No response..................................	→ **P214**
**P213**	What family planning methods do you use?<br><br>**YOU MAY MARK MORE THAN ONE OPTION**	- ( ) Birth Control Pills<br>- ( ) Injectable methods<br>- ( ) Intrauterine Devices<br>- ( ) Condoms<br>- ( ) Surgical sterilization<br>- ( ) Natural methods (Billings, temperature or calendar)<br>- ( ) Other /Specify ________________<br><br>0- ( ) No response	
**If the response was YES to question P212 →GO TO SECTION 3**			
**P214**	What are the reasons not to use family planning methods?	1- ( ) You want to get pregnant<br>2- ( ) You don't know where to get them<br>3- ( ) You don't find it necessary<br>4- ( ) Your partner prohibits the use<br>5- ( ) You don't like it<br>6- ( ) Other / Specify_________________<br>0- ( ) No response	

SECTION 3: KNOWLEDGE, OPINIONS AND ATTITUDES REGARDING HIV

We will be asking questions of your knowledge and opinions about HIV.

No.	Questions	Categories	Go to
**P301**	What can a woman who is HIV+ that is pregnant do to prevent transmission of HIV to her baby?<br><br>**YOU MAY MARK MORE THAN ONE OPTION**	- ( ) Start medical control<br>- ( ) Take antiretroviral medication<br>- ( ) Ask for a caesarean<br>- ( ) Don't Breast feed<br>- ( ) Other/Specify ________________<br>3- ( ) Does not know<br>0- ( ) No response	
**P302**	Do you think a person with HIV can be re-infected, meaning that although the person has been diagnosed once with HIV can the person be re-infected?	1- ( ) Yes<br>2- ( ) No...<br>3- ( ) Does not know..<br>0- ( ) No response...	→**Secc. 4**
**P303**	Do you think that using condoms consistently and correctly for every sexual act can prevent re-infection of HIV?	1- ( ) Yes<br>2- ( ) No<br>3- ( ) No does not know<br>0- ( ) No response	
**P304**	Do you think being faithful to one sexual partner can prevent the re-infection of HIV?	1- ( ) Yes<br>2- ( ) No<br>3- ( ) Does not know<br>0- ( ) No response	
**P305**	Do you consider that your risk of re-infection with HIV is:	1- ( ) High<br>2- ( ) Medium<br>3- ( ) Low<br>4- ( ) No risk<br>5- ( )Does not know<br>0- ( ) No response	
**P306**	What can people with HIV do to prevent re-inf ection?	1- ( ) Use condoms in all sexual acts<br>2- ( ) Don't have sex<br>3- ( ) Can't do anything<br>4- ( ) Other/Specify_________________<br>5-( ) Does not know<br>0- ( ) No response	

SECTION 4 CONDOMS

We will be asking questions concerning Condoms

No.	Questions	Categories	Go to
**P401**	What places or people do you know where you can get condoms?<br><br>**YOU MAY MARK MORE THAN ONE OPTION**	- ( ) Supermarket / Shop<br>- ( ) Pharmacy<br>- ( ) Health Center / Hospital<br>- ( ) NGO<br>- ( ) Night Club/Bar/Disco<br>- ( ) Brothel / Motel<br>- ( ) Other/Specify__________________<br><br>0- ( ) No response	
**P402**	For you getting condoms is …?<br><br>**READ THE OPTIONS**	1- ( ) Very easy……………………………………<br>2- ( ) Easy……………………………………<br>3- ( ) Difficult……………………………………<br>4- ( ) Very difficult ……………………………<br><br>0- ( ) No response……………………………	➐ **P403**<br><br>➐ **P404**<br><br>➐ **P405**
**P403**	Why is it easy to get a condom?<br><br>**READ OPTIONS**<br><br>**YOU MAY MARK MORE THAN ONE OPTION**	- ( ) I get them for free<br>- ( ) They are cheap<br>- ( ) The place where I buy them is close by<br>- ( ) I am not ashamed to buy them<br>- ( ) Other/ Specify ________________<br><br>0- ( ) No response	
		➐ **GO TO P405**	
**P404**	Why is it difficult to get a condom?<br><br>**READ OPTIONS**<br><br>**YOU MAY MARK MORE THAN ONE OPTION**	- ( ) I feel ashamed to purchase them<br>- ( ) Don't know where to get them<br>- ( ) Don't have Money to buy them<br>- ( ) When i go get there are none<br>- ( ) they are too expensive<br>- ( ) Other/ Specify_________________<br><br>0- ( ) No response	
**P405**	In the last week how many condoms have you received for f ree?	Number of free condoms: ______<br><br>0- ( ) No response	
**P406**	What places or people do you know where you can get lubricant?<br><br>**YOU MAY MARK MORE THAN ONE OPTION**	- ( ) Supermarket / Shop<br>- ( ) Pharmacy<br>- ( ) Health Center / Hospital<br>- ( ) NGO<br>- ( ) Night Club/Bar/Disco<br>- ( ) Brothel / Motel<br>- ( ) Other /Specify__________________<br><br>0- ( ) No response	

**P407**	Do you consider getting lubricants to be…?	1- ( ) Very Easy<br>2- ( ) Easy<br>3- ( ) Difficult<br>4- ( ) Very difficult<br>0- ( ) No response	

No.	Questions	Categories	Go to
**P408**	Do you f requently use lubricant during sex?	1- ( ) Yes<br>2- ( ) No………………………………………………<br>0- ( ) No response…………………………………… }	➔ **411**
**P409**	What type of lubricant do you use during sexual intercourse?<br><br>**READ THE OPTIONS**<br><br>**YOU MAY MARK MORE THAN ONE OPTION**	- ( ) Water based lubricants<br>- ( ) Vaseline<br>- ( ) Skin or Hand creams<br>- ( ) Vaginal Gel<br>- ( ) Baby Oil<br>- ( ) Butter<br>- ( ) Cooking Oil<br>- ( ) Saliva<br>- ( ) Other/Specify___________________<br><br>0- ( ) No response	
**P410**	Do you have lubricants at this moment?	1- ( ) Yes<br>2- ( ) No<br>0- ( ) No Response	
**P411**	When you are provided condoms, are you also provided with lubricants?	1- ( ) Yes<br>2- ( ) No<br>0- ( ) No response	

SECTION 5: HEALTH SITUATION

In this section I am going to ask you some questions about your health status and HIV testing. Remember that your responses will remain confidential.

No.	Questions	Categories	Go to
**P501**	How long ago did you receive your HIV diagnosis?	1- ( ) Less than 1 month<br>2- ( ) From 1 to 5 months<br>3- ( ) From 6 to 11 months<br>4- ( ) From 1 to 2 years<br>5- ( ) From 3 to 5 years<br>6- ( ) From 6 to 10 years<br>7- ( ) More than 10 years<br>0- ( ) No response	
**P502**	When you learned about your HIV diagnosis, what was the reason you decided to get tested?	1- ( ) You did it voluntarily<br>2- ( ) It was requested<br>3- ( ) Because you were sick<br>4- ( ) Because your partner was diagnosed with HIV/AIDS<br>5- ( ) While donating Blood<br>6- ( ) Other/Specify_____________<br>0- ( ) No response	

**P503**	Did you get counseling when you were tested for HIV?<br><br>**READ ALL OPTIONS**	1- ( ) Yes, I received it before the test<br>2- ( ) Yes, upon receiving the result of the test<br>3- ( ) Yes, prior to receiving the result and after receiving the test<br>4- ( ) No I did not receive counseling<br>0- ( ) No response	
**P504**	Where did you get tested for HIV?	1- ( ) Health Centre / Public Hospital<br>2- ( ) Social Security (VCT)<br>3- ( ) Clinic / Private Laboratory<br>4- ( ) Blood bank<br>5- ( ) Red Cross<br>6- ( ) NGO<br>7- ( ) Other/Specify_____________<br>0- ( ) No response	
**P505**	Are you currently attending medical supervision for being a person living with HIV?	1- ( ) Yes<br>2- ( ) No...<br>0- ( ) No response................................	➔ **P509**
**P506**	How often do you have to go for medical control because of you HIV diagnosis?	1- ( ) From two weeks or less<br>2- ( ) From 3 weeks to a month<br>3- ( ) Every 2 to 3 months<br>4- ( ) Every 4 to 5 months<br>5- ( ) Every 6 months<br>0- ( ) No response	
**P507**	And in practice really how often will you attend your medical control?	1- ( ) 2 weeks or less<br>2- ( ) From 3 weeks to a month<br>3- ( ) 2 or 3 months<br>4- ( ) Every 4 to 5 months<br>5- ( ) Every 6 months<br>0- ( ) No response	
**No.**	**Question**	**Categories**	**Go to**
**P508**	What kind of services do you receive when attending medical control for being a person with HIV?<br><br>**YOU MAY MARK MORE THAN ONE OPTION**	- ( ) Medical consultation<br>- ( ) Antiretroviral therapy<br>- ( ) Treatment for Opportunistic Infections<br>- ( ) Laboratory tests<br>- ( ) Special tests (CD4 and Viral Load)<br>- ( ) Counseling and education<br>- ( ) Nutritional counseling<br>- ( ) Dental Services<br>- ( ) Psychological Services<br>- ( ) Sexual and reproductive services<br>- ( ) Other/Specify______________<br>0- ( ) No response	
**P509**	Do you consider that after knowing your HIV status, do you take care less, equal or more?	1- ( ) Less<br>2- ( ) Same<br>3- ( ) More<br>0- ( ) No response	

**P510**	Which of the following do you regularly do for you to take care of your health?<br><br>**PROBE FOR EACH OPTION AND CHECK THE ONE THE PERSON DOES**	- ( ) Use a condom every time you have sex<br>- ( ) Use condoms with people who you don't know<br>- ( ) Use a condom only with your stable partner (s)<br>- ( ) Use condoms sometimes<br>- ( ) Does not have sex with strangers<br>- ( ) You do not have vaginal intercourse<br>- ( ) You do not have anal intercourse<br>- ( ) You do not have oral sex<br>- ( ) Practice abstinence (does not have sex of any kind)<br>- ( ) Practice mutual fidelity with your partner<br>- ( ) You have reduced the number of sexual partners<br>- ( ) You Attend counselling<br>- ( ) You Assist medical services when you have appointments<br>- ( ) You are a member of a support group<br>- ( ) You don't consume alcohol<br>- ( ) You don't consume drugs<br>- ( ) You have improved your diet<br>- ( ) You take the medications that have been prescribed<br>- ( ) You practice hobbies<br>- ( ) You exercise regularly<br>- ( ) You have social support networks<br>- ( ) Other /Specify______________<br>0- ( ) No response	
**THE FOLLOWING QUESTIONS ARE ONLY FOR THOSE WHO SAID THAT THEY TAKE MORE CARE OF THEIR HEALTH IN QUESTION P509**			
**No.**	**Questions**	**Categories**	**Go to**
**P511**	What motivated you to take action and take care of your health?<br><br>**YOU MAY MARK MORE THAN ONE OPTION**	- ( ) The radio messages<br>- ( ) Television messages<br>- ( ) written information you have read<br>- ( ) Conversations with my friends<br>- ( ) The talks received from NGO's<br>- ( ) Your religious Beliefs<br>- ( ) The death of someone close<br>- ( ) Love for your children, family or partner<br>- ( ) Self-esteem<br>- ( ) Attending support group meetings<br>- ( ) I want to live<br>- ( ) Home Visit strategies<br>- ( ) Other /Specify______________<br><br>0- ( ) No response	
**P512**	Have you had any health problems or infection in the past 3 months?	1- ( ) Yes<br>2- ( ) No<br>0- ( ) No response	

No.	Questions	Categories	Go to
**P513**	Have you had any of the following health problems or infection in the past 3 months?<br><br>**PROBE FOR EACH OPTION AND CHECK THE ONE THE PERSON DOES**	- ( ) Fever for more than 15 days<br>- ( ) Diarrhea for more than 3 days<br>- ( ) General illnesses<br>- ( ) Cough for more than 15 days<br>- ( ) Skin abscesses<br>- ( ) Sexually transmitted infections<br>- ( ) Swollen lymph<br>- ( ) Skin diseases<br>- ( ) Seizures<br>- ( ) Persistent headache<br>- ( ) Oral candidiasis<br>- ( ) Pneumonia<br>- ( ) Tuberculosis<br>- ( ) Other /Specify______________<br>3- ( ) None<br>0- ( ) No response	
**IF YOU HAVE NOT HAD ANY SICKNESS → Go to P519**			
**P514**	The last time you presented this health issue what did you do?<br><br>**YOU MAY MARK MORE THAN ONE OPTION**	- ( ) You consulted for health service at the center you attend<br>- ( ) You consulted a private physician<br>- ( ) You self-medicated<br>- ( ) Other /Specify_____________<br>3- ( ) You did nothing……………………<br>0- ( ) No response………………………..	**→P521**
**No.**	**Questions**	**Categories**	**Go to**
**P515**	How long after the f irst symptoms appeared did you f ind help at a Health Centre, Clinic, Hospital, or NGO?	1- ( ) 1 week or less<br>2- ( ) More than 1 week but less than a month<br>3- ( ) 1 month or more<br>4- ( ) Did not seek help.......................<br>0- ( ) No response...............................	**→P521**
**P516**	Did you get prescription drugs?	1- ( ) Yes<br>2- ( ) No...<br>0- ( ) No response...............................	**→P521**
**P517**	Did the medical center where you consulted provide the medication?<br><br>**READ OPTIONS**	1- ( ) Yes, they provided everything that was needed...............................<br>2- ( ) They provided some but not all<br>3- ( ) They did not provide any of the medications<br>0- ( ) No response...............................	**→P519**<br><br>**→P519**
**P518**	Did you get the prescription elsewhere?	1- ( ) Yes, got everything I needed<br>2- ( ) Got some but not all<br>3- ( ) Did not get the medication<br>0- ( ) No response	

**P519**	Did you take or apply the prescribed doses?	1- ( ) Yes...<br>2- ( ) No<br>0- ( ) No response...............................	→P521<br><br>→P521
**P520**	Why did you not take the full dose?<br><br>**YOU MAY MARK MORE THAN ONE OPTION**	- ( ) The symptoms disappeared<br>- ( ) You felt better<br>- ( ) The medication made you feel bad<br>- ( ) You were not sure how to take or apply the medication<br>- ( ) You had no money for medicine<br>- ( ) You forgot<br>0- ( ) No response	
**P521**	Would you say that your current health is?	1- ( ) Very good<br>2- ( ) Good<br>3- ( ) Stable<br>4- ( ) Bad<br>5- ( ) Very bad<br>0- ( ) No response	
**P522**	In the last 12 months, have you had to pay for health services related to your diagnosis within the public service?	1- ( ) Yes<br>2- ( ) No...<br>0- ( ) No response................................ }	→P524
**No.**	**Questions**	**Categories**	**Go to**
**P523**	What services did you have to pay?<br><br>**PROBE FOR EACHESERVICE AND MARK THOSETHAT THE PERSON HAD TO PAY**	- ( ) General Consult<br>- ( ) Consultation for a specialist<br>- ( ) Laboratory tests<br>- ( ) Medications for OI<br>- ( ) ARV Medication<br>- ( ) Other /Specify_____________<br>0- ( ) No response	
**P524**	In the last 12 months have you had to pay for private health services?	1- ( ) Yes<br>2- ( ) No...<br>0- ( ) No response................................ }	➐ P52 6
**P525**	Which private services have you had to pay?<br><br>**PROBE FOR EACHESERVICE AND MARK THOSETHAT THE PERSON HAD TO PAY**	- ( ) General Consult<br>- ( ) Consultation for a specialist<br>- ( ) Laboratory Tests<br>- ( ) Medications for OI<br>- ( ) ART Medication<br>- ( ) Other /Specify_____________<br>0- ( ) No response	
**P526**	Are you taking ARVs / ARTs	1- ( ) Yes<br>2- ( ) No..<br>0- ( ) No response.............................. }	➐ P53 8
**P527**	How does your health service provide your antiretroviral drugs?<br><br>**READ OPTIONS**	1- ( ) Complete and Free<br>2- ( ) Only Complete<br>3- ( ) Only Free<br>4- ( ) Neither complete nor free<br>0- ( ) No response	
**P528**	How long ago did you start taking your Antiretroviral Medications?	1- ( ) Less than 1 month<br>2- ( ) From 1 to 5 months<br>3- ( ) From 6 to 11 months<br>4- ( ) From 1 or 2 years<br>5- ( ) From 3 to 5 years	

		6- ( ) From 5 to 10 years<br>7- ( ) More than 10 years<br>0- ( ) No response	
**P529**	Which of the following people give you the attention and control your antiretroviral therapy?<br><br>**PROBE FOR EACH MEDICAL PROFESSIONAL AND MARK THOSE THAT HAVE ATTENDED THE PERSON BEING INTERVIEDED**	- ( ) Medical Specialist<br>- ( ) General Practitioner<br>- ( ) Nurse<br>- ( ) Counselor<br>- ( ) Other /Specify______________<br><br>0- ( ) No response	

No.	Questiones	Categories	Go to
**P530**	Which of the following antiretroviral medications are you currently taking?	- ( ) Abacavir (Ziagen, Virol y Abavir)<br>- ( ) Abacavir + Lamivudina (Kivexa)<br>- ( ) Abacavir+ Lamivudina+ Zidovudina (Trizivir y Triviro)<br>- ( ) Atazanavir (Reyataz)<br>- ( ) Darunavir (Prezista)<br>- ( ) Delavirdina<br>- ( ) Didodasina (Videx y Didex)<br>- ( ) Efavirenz (Sustiva, Aviranz y Efavir)<br>- ( ) Elvitegravir<br>- ( ) Emtricitabina (Emtriva)<br>- ( ) Emtricitabina/Tenofovir (Truvada)<br>- ( ) Emtricitabina/Tenofovir/Efavirez (Atripla ó Viraday).<br>- ( ) Enfuvirtide (Fuzeon)<br>- ( ) Estavudina<br>- ( ) Etravirina<br>- ( ) Fosamprenavir<br>- ( ) Indinavir (Crixivan)<br>- ( ) Lamivudina (Epivir, Lamivir ó 3TC)<br>- ( ) Lamivudina / Estavudina / Nevirapina (Triomune 40)<br>- ( ) Lopinavir<br>- ( ) Lopinavir/ritonavir (Kaletra, Kameltrex ó Aluvia)<br>- ( ) Maraviroc<br>- ( ) Nevirapina (Viramune, Nevimune o Nevipan)<br>- ( ) Raltegravir<br>- ( ) Ritonavir (Norvir)<br>- ( ) Saquinavir (Invirase)<br>- ( ) Tenofovir (Tenovir, Tenvir)<br>- ( ) Tipranavir<br>- ( ) Zidovudina (Retrovir, Zidovir ó AZT)<br>- ( ) Zidovudina + Lamivudina (Combivir / Duovir)<br>0- ( ) No Response	
**P531**	In the last 12 months have you had adverse effects because of the antiretroviral therapy?	1- ( ) Yes<br>2- ( ) No..	**❼P534**

No.	Questions	Categories	Go to
		0- ( ) No Response.................................	
**P532**	What did you do when the adverse effects were presented caused by the antiretroviral therapy?	1- ( ) Consulted the doctor in the health center<br>2- ( ) Consulted a private doctor<br>3- ( ) Went to an NGO<br>4- ( ) Consulted with a friend or relative<br>5- ( ) Consulted with the counselor or health provider<br>6- ( ) Other /Specify ____________<br>7- ( ) Suspended ART's without consulting anyone ……………<br>8- ( ) Did nothing ....<br>0- ( ) No response.................................	 <br><br><br><br><br><br>➐ **P534**<br><br>➐ **P534**
**P533**	What consequences did the query have?	1- ( ) They changed the scheme ART<br>2- ( ) They kept the scheme ART<br>0- ( ) No response	
**P534**	Since you began attending ART, have you ever suspended treatment by your own choice? That is, by your own will and not for reasons of medical prescription?	1- ( ) Yes<br>2- ( ) No..<br>0- ( ) No response................................	 ➐ **P536**<br>➐ **P536**
**P535**	What are the reasons that you have decided to stop taking your antiretroviral medication?<br><br>**YOU MAY MARK MORE THAN ONE OPTION**	- ( ) Because of the cost<br>- ( ) They cause side effects<br>- ( ) It's difficult to remember<br>- ( ) Its difficult / complicated taking them<br>- ( ) I have missed my medical appointments<br>- ( ) Because of fatigue<br>- ( ) On recommendation of my church<br>- ( ) Other /Specify ______________<br>0- ( ) No response	
**P536**	In the last 12 months, have forgotten at least once to go for your antiretroviral medication at the health center?	1- ( ) Yes<br>2- ( ) No<br>0- ( ) No response	
**P537**	In the last 12 months, has the health center stopped supplying you with antiretroviral medication?	1- ( ) Yes<br>2- ( ) No<br>0- ( ) No response	
**P538**	When a person with HIV is taking antiretroviral medication, does the person get sick less, the same or more than if they are not taking ART's?	1- ( ) Get sick less<br>2- ( ) Get sick the same<br>3- ( ) Gets more sick<br>4- ( ) Does not know<br>0- ( ) No response	

**P539**	And when a person with HIV is taking antiretroviral medication, does the person live less, the same or longer than if not taking ARTs?	1- ( ) Lives less<br>2- ( ) Lives the same<br>3- ( ) Lives more<br>4- ( ) Does not know<br>0- ( ) No response	

No.	Questions	Categories	Go to
**P540**	And by taking the antiretroviral medication, do you have less, the same or more likely to transmit HIV?	1- ( ) Less probability<br>2- ( ) Same probability<br>3- ( ) More probability<br>4- ( ) Does not know<br>0- ( ) No response	
**P541**	Have you done a CD4 test in the last 12 months?	1- ( ) Yes<br>2- ( ) No...<br>0- ( ) No response................................	544
**P542**	Do you know the results of the latest CD4?	1- ( ) Yes<br>2- ( ) No..<br>0- ( ) No response................................	544
**P543**	What was your CD4 count?	1- ( ) Less than 100<br>2- ( ) From 101 to 300<br>3- ( ) From 301 to 500<br>4- ( ) From 501 to 750<br>5- ( ) From 750 to 1000<br>6- ( ) More than 1000<br>0- ( ) No response	
**P544**	In the past 12 months have you done a Viral Load Test?	1- ( ) Yes<br>2- ( ) No...<br>0- ( ) No response................................	547
**P545**	Do you know the results of your last Viral Load Test?	1- ( ) Yes<br>2- ( ) No..<br>0- ( ) No response................................	547
**P546**	What was the result of the Viral Load?	1- ( ) Undetectable<br>2- ( ) Less than 10000<br>3- ( ) More than 10000	
**P547**	After you found out that you are HIV $^{+}$, have you received treatment to prevent tuberculosis?	1- ( ) Yes<br>2- ( ) No<br>0- ( ) No response	
**P548**	Being a person with HIV, have you ever been ill with tuberculosis?	1- ( ) Yes<br>2- ( ) No...<br>0- ( ) No response................................	Secc. 6
**P549**	Being a person with HIV, have you received treatment to cure tuberculosis?	1- ( ) Yes<br>2- ( ) No<br>0- ( ) No response	

SECTION 6: DISCRIMINATION, STIGMA, AND SUPPORT TO RIGHTS

By discrimination to people with HIV it is understood that it's the social behavior that separates, isolates, and are considered less than other people because of the diagnosis, in any area of our daily life or upon requesting services to which we are entitled.

It is necessary to emphasize that many people receive some form of abuse in their work, school, family or requesting or receiving a service. We ask that you endeavor to refer if in your case, you have lived a discriminatory situation or abuse solely on you status as a person with HIV.

No.	Questions	Categories	Go to
**P601**	Have you ever been the victim of some type of abuse in the health services for being a person with HIV?	1- ( ) Yes<br>2- ( ) No<br>0- ( ) No response	
**P602**	When you receive medical services, do you consider it necessary to hide that you are a person with HIV?	1- ( ) Yes<br>2- ( ) No<br>0- ( ) No response	
**P603**	Have you ever been forced, for work purposes, to do an HIV test?	1- ( ) Yes<br>2- ( ) No<br>0- ( ) No response	
**P604**	Does your family know you are a person with HIV?	1- ( ) Yes<br>2- ( ) No..<br>0- ( ) No response...............................	**→P606**
**P605**	What did your family do when they learned of your diagnosis?	1- ( ) They supported me<br>2- ( ) They rejected me<br>4- ( ) They abandoned me<br>5- ( ) They kicked me out<br>6- ( ) Other/Specify_______________<br>0- ( ) No response	
**P606**	To find work did you find the need to hide that you are person with HIV?	1- ( ) Yes<br>2- ( ) No<br>0- ( ) No response	
**P607**	Do you have someone who can accompany you to the doctor or hospital, if you need it?	1- ( ) Yes<br>2- ( ) No<br>0- ( ) No response	
**P608**	Would you denounce an act of violation of your rights?	1- ( ) Yes..<br>2- ( ) No<br>3-( ) does not know...................................<br>0- ( ) No response................................	**→Secc.7**<br><br>**→Secc.7**
**P609**	Why would you not denounce an act of violation of your rights?<br><br>**YOU MAY MARK MORE THAN ONE OPTION**	- ( ) It would not change anything<br>- ( ) They would realize that I am a person with HIV<br>- ( ) To protect my family<br>- ( ) Don't know how to do it<br>- ( ) Don't have time<br>- ( ) Other / Specify_______________<br>0- ( ) No response	

SECTION 7: ALCOHOL AND DRUG USE

Now I will ask you some questions about alcohol and drug use.

No.	Questions	Categories	Go to
**P701**	Have you taken any alcohol or liquor during the past month?	1- ( ) Yes<br>2- ( ) No...<br>0- ( ) No response................................	→P703
**P702**	Over the past month, on average how many drinks or bottles of beer did you consume per week?	Number of drinks _________<br><br>0- ( ) No response	
**P703**	Considering that we always refer to those drugs that are not used by prescription for treatment in the last year have you....... ?<br><br>**PROBE FOR EACH DRUG AND MARK THOSE THAT THE HAS CONSUMED**	- ( ) Smoked marijuana?<br>- ( ) Smoked Rock or crack?<br>- ( ) Inhaled Rock or crack?<br>- ( ) Inhaled cocaine?<br>- ( ) Injected cocaine?<br>- ( ) Injected heroin?<br>- ( ) Inhaled thinner or glue?<br>- ( ) consumed ecstasy?<br>- ( ) Taken Amphetamines?<br>- ( ) Taken Diazepam?<br>- ( ) Other /Specify____________<br>3- ( ) None<br>0- ( ) No response	
**IF NO DRUG IS MENTIONED GO TO SECTION 8**			
**P704**	Thinking of those drugs that are not used by prescription for treatment, during the last month how many times have you used the drug you consumed the most?	Number of times _________<br><br>0- ( ) No response	
**THE NEXT QUESTION IS ONLY IF YOU HAVE INDICATED IN P703 THAT YOU INJECTED ANY DRUG IF NO, GO TO SECTION 8**			
**P705**	When injecting drugs, did another person inject before or after you with the same syringe, needle or other injection equipment?	1- ( ) Yes<br>2- ( ) No<br>3- ( ) Does not know<br>0- ( ) No response	

SECTION 8: SEX WITH STABLE PARTNERS

We will ask you about sexual intercourse with regular partners, meaning steady partner (s) to people you had sex that were not paid and which maintains an affective / constant / regular relationship, such as your spouse, boyfriend or husband / wife, but do not live in the same house.

No.	Questions	Categories	Go to
P801	Currently do you have a Stable partner?	1- ( ) Yes<br>2- ( ) No..<br>0- ( ) No response................................	→Secc.9
P802	Is your stable partner a person with HIV?	1- ( ) Yes<br>2- ( ) No<br>0- ( ) No response	
P803	Does your stable partner know that you are a person with HIV?	1- ( ) Yes<br>2- ( ) No<br>0- ( ) No response	
P804	The last time you had sex with a steady partner did you use a condom?	1- ( ) Yes<br>2- ( ) No..<br>0- ( ) No response................................	➐P806
P805	Who suggested condom use the last time you had sex?	1- ( ) I did<br>2- ( ) My Partner<br>3- ( ) Joint decision<br>0- ( ) No response	
**IF USED CONDOM IN YOUR LAST RELATIONSHIP (P804) ➐ GO TO P807**			
P806	Why did you and your stable partner, not use a condom the last time you had sex?<br><br>**YOU MAY MARK MORE THAN ONE OPTION**	- ( ) I trust my partner<br>- ( ) My partner did not want to use<br>- ( ) I don't like to use it<br>- ( ) I was under the inf luence of alcohol or drugs<br>- ( ) Did not have a condom at that time<br>- ( ) Condoms are very expensive<br>- ( ) Condoms don't work<br>- ( ) Other /Specify____________<br><br>0- ( ) No response	
P807	In the past year, how often did you use condoms with your stable sexual partner?	1- ( ) All the times<br>2- ( ) Most of the times<br>3- ( ) Often<br>4- ( ) Few times<br>5- ( ) almost never<br>6- ( ) Never<br>0- ( ) No response	
P808	The last time you had sex with a stable partner with a condom; did you use lubricant all the time of penetration?	1- ( ) Yes<br>2- ( ) No<br>0- ( ) No response	
P809	Do you and your partner usually use lubricant during sex?	1- ( ) Yes<br>2- ( ) No..<br>0- ( ) No response................................	→Secc.9

No.	Questions	Categories	Go to
P810	In the last month, how often did you use lubricant with your stable sexual partner?	1- ( ) All the time<br>2- ( ) Most of the time<br>3- ( ) Often<br>4- ( ) Few Time<br>5- ( ) Almost never<br>6- ( ) Never<br>0- ( ) No response	

SECTION 9: SEXUAL RELATIONS WITH OCCASIONAL PARTNERS

We will ask you about sex with occasional partners, understanding that these people you had sex with did not pay and that you do not maintain a stable, constant or regular relationship.

Remember that all your answers will maintain confidentiality and anonymity.

No.	Questions	Categories	Pase a
P901	In the last year have you had sex with an occasional partner?	1- ( ) Yes<br>2- ( ) No..<br>0- ( ) No response................................	→Secc.10
P902	The last time you had sex with an occasional partner did you use a condom?	1- ( ) Yes<br>2- ( ) No..<br>0- ( ) No response................................	❼P904
P903	Who suggested condom use the last time you had sex?	1- ( ) I did<br>2- ( ) My partner<br>3- ( ) Joint decision<br>0- ( ) No response	
IF YOU USED A CONDOM IN YOUR LAST RELATIONSHIP (P902)❼ GO TO P905			
P904	Why did you not use a condom the last time you had sex?<br><br>**YOU MAY MARK MORE THAN ONE OPTION**	- ( ) I trust my partner<br>- ( ) My partner did not want to use<br>- ( ) I don't like to use it<br>- ( ) I was under the inf luence of alcohol or drugs<br>- ( ) Did not have a condom at that moment<br>- ( ) Condoms are very expensive<br>- ( ) Condoms do not work<br>- ( ) Other /Specify____________<br>0- ( ) No response	
P905	The last time you had sex with an occasional partner did you use lubricant during penetration?	1- ( ) Yes<br>2- ( ) No<br>0- ( ) No response	
P906	Think about your sexual encounters with occasional partners, how often did you use condom?	1- ( ) All the time<br>2- ( ) Most of the times<br>3- ( ) Often<br>4- ( ) Few times<br>5- ( ) Almost never<br>6- ( ) Never	

		0- ( ) No response	
**P907**	The last occasional partner whom you had sex has HIV?	1- ( ) Yes<br>2- ( ) No<br>3- ( ) Does not know<br>0- ( ) No response	
**P908**	Do you inform your occasional partners that you are person with HIV?	1- ( ) Yes<br>2- ( ) No<br>0- ( ) No response	
**No.**	**Questions**	**Categories**	**Go to**
**P909**	What are the places you f requently go to has for sexual encounters with occasional partners?<br><br>**YOU MAY MARK MORE THAN ONE OPTION**	- ( ) Motels<br>- ( ) Bars<br>- ( ) Parks<br>- ( ) Darkrooms<br>- ( ) Saunas<br>- ( ) Massage rooms<br>- ( ) Cinemas<br>- ( ) Shopping Centers<br>- ( ) public bathrooms<br>- ( ) Brothels<br>- ( ) Other /Specify____________<br>0- ( ) No response	
**P910**	The last sexual encounter with an occasional partner, was with a known or unknown person?	1- ( ) Known<br>2- ( ) Unknown<br>0- ( ) No response	

SECTION 10: SEX WITH SEX WORKERS

We will now ask you about sex with sex workers.

No.	Questions	Categories	Go to
**P1001**	In the last year have you had sex with a sex worker?	1- ( ) Yes<br>2- ( ) No...<br>0- ( ) No response...............................	→Secc.11
**P1002**	In the last year which sexual service have you purchased?<br><br>**YOU MAY MARK MORE THAN ONE OPTION**	- ( ) Anal Sex<br>- ( ) Vaginal Sex<br>- ( ) Oral Sex<br>- ( ) Masturbation<br>- ( ) Other / Specify____________<br>0- ( ) No response	
**P1003**	The last time you paid to have sex, did you use a condom?	1- ( ) Yes...<br>2- ( ) No<br>0- ( ) No response...............................	➐P1005<br><br>➐P1005
**P1004**	Why did you not use a condom the last time you paid for sex?<br><br>**YOU MAY MARK MORE THAN ONE OPTION**	- ( ) I trust my partner<br>- ( ) My partner did not want to use<br>- ( ) I do not like to use<br>- ( ) Was under the influence of alcohol or drugs<br>- ( ) Did not have a condom at the time	

		- ( ) Condoms are very expensive<br>- ( ) Condoms do not work<br>- ( ) Other /Specify____________<br>0- ( ) No response	
**P1005**	Over the past year, how often did you use condoms, the times that you paid for sex?	1- ( ) All the times<br>2- ( ) Most of the times<br>3- ( ) Often<br>4- ( ) Few times<br>5- ( ) Almost Never<br>6- ( ) Never<br>0- ( ) No response	
**P1006**	The last time you paid for sex, did you use lubricant during penetration?	1- ( ) Yes<br>2- ( ) No<br>0- ( ) No response	
**P1007**	Do you inform the sex workers that you are person with HIV?	1- ( ) Yes<br>2- ( ) No<br>0- ( ) No response	
**P1008**	Of the people with whom you have had paid sex do you know if they are HIV^+?	1- ( ) Yes<br>2- ( ) No<br>3- ( ) Does not know<br>0- ( ) No response	

SECTION 11. LAST SEXUAL RELATIONSHIP

Now I will ask questions about your last sexual encounter.

No.	Questions	Categories	Go to
**P1101**	Think about your last sexual encounter with what kind of partner did you have your last sexual intercourse?<br><br>**READ OPTIONS**	1- ( ) Stable partner, live with him/her<br>2- ( ) Stable partner, don't live with him/her<br>3- ( ) Client<br>4- ( ) Occasional partner<br>5- ( ) Commercial partner<br>6- ( ) Unknown person<br>0- ( ) No response	
**P1102**	Did you use a condom the last time you had sexual relations?	1- ( ) Yes<br>2- ( ) No……………………………………<br>0- ( ) No response……………………	} ➐**P1104**
**P1103**	Who suggested using a condom in your last sexual intercourse?	1- ( ) I did<br>2- ( ) My partner<br>3- ( ) Joint decision<br>0- ( ) No response	
**IF YOU USED A CONDOM IN YOUR LAST RELATIONSHIP (P1102) ➐ GO TO P1105**			
**P1104**	Why did you not use a condom the last time you had sex?	- ( ) I trust my partner<br>- ( ) My partner did not want to use<br>- ( ) I do not like to use	

	**YOU MAY MARK MORE THAN ONE OPTION**	- ( ) Was under the influence of alcohol or drugs<br>- ( ) Did not have a condom at the time<br>- ( ) Condoms are very expensive<br>- ( ) Condoms do not work<br>- ( ) Other /Specify____________<br><br>0- ( ) No response	
**P1105**	This last time you had sex did you use lubricant during penetration?	1- ( ) Yes<br>2- ( ) No<br>0- ( ) No response	
**P1106**	Is the person you had sex with a person with HIV?	1- ( ) Yes<br>2- ( ) No<br><br>3- ( ) Does not know<br>0- ( ) No response	
**P1007**	Did you tell the person you had sex with that you are a person with HIV?	1- ( ) Yes<br>2- ( ) No<br><br>0- ( ) No response	
**No.**	**Questions**	**Categories**	**Go to**
**P1108**	Considering all sexual partners during the last month ...<br><br>A. Have you ever had sex without using a condom?<br><br>B. Have you ever had anal intercourse (or have you been penetrated or have you penetrated f rom behind) not using a condom?<br><br>C. Has a man ejaculated in your mouth without a condom?<br><br>D. Did you use lubricant in all your sexual acts?	A.<br>1- ( ) Yes<br>2- ( ) No<br>0- ( ) No response<br><br>B.<br>1- ( ) Yes<br>2- ( ) No<br>0- ( ) No response<br><br>C.<br>1- ( ) Yes<br>2- ( ) No<br>0- ( ) No response<br><br>D.<br>1- ( ) Yes<br>2- ( ) No<br>0- ( ) No response	

SECTION 12: SEX WORK

Now I would like to ask you some personal questions about sex work, sex worker is understood as receiving payment for sex with money, food, clothing, jobs or other favors.

Remember that your answers will maintain confidential and anonymous.

No.	Questions	Categories	Go to
**P1201**	In the last 12 months have you done sex work?	1- ( ) Yes<br>2- ( ) No...<br>0- ( ) No response................................	→**Secc.13**
**P1202**	How old were you when you were f irst paid for sex	Age _____<br>00- ( ) No response	
**P1203**	Do you tell your clients that you have HIV?	1- ( ) Yes<br>2- ( ) No<br>0- ( ) No response	
**P1204**	During the last year have you been to other cities to do sex work?	1- ( ) Yes<br>2- ( ) No<br>0- ( ) No response	
**P1205**	During the last year have you been to another country for sex work?	1- ( ) Yes<br>2- ( ) No<br>0- ( ) No response	
**P1206**	During the last year which has been the main area or place for sex work, i.e. where you get your client more often?<br>**READ THE OPTIONS**	1- ( ) Brothel / Business<br>2- ( ) Brothel<br>3- ( ) Road / Street / Park<br>4- ( ) Bar / Night Club<br>5- ( ) Motel / Hotel / Lodging<br>6- ( ) Massage Parlor<br>7- ( ) Phone or Internet<br>8- ( ) Other /Specify________________<br>0- ( ) No response	
**P1207**	In general, how many clients do you service in a week?	Number of clients __________<br>0- ( ) No response	
**P1208**	The last time you had sex with a client did you use a condom?	1- ( ) Yes<br>2- ( ) No...<br>0- ( ) No response................................	→ **P1210**
**P1209**	Who suggested condom use that time?	1- ( ) Me<br>2- ( ) My client<br>3- ( ) Joint decision<br>0- ( ) No response	

IF A CONDOM WAS USED IN LAST RELATIONSHIP (P1208) → GO TO P1211

No.	Questions	Categories	Go to
**P1210**	Why did you and your client not use a condom that last time?<br><br>**YOU MAY MARK MORE THAN ONE OPTION**	- ( ) The client paid more<br>- ( ) I think he/she is healthy<br>- ( ) He did not want to use<br>- ( ) I do not like to use<br>- ( ) I was under the inf luence of alcohol or drugs<br>- ( ) Did not have a condom at the time<br>- ( ) Condoms are very expensive<br>- ( ) Condoms do not work<br>- ( ) The condom had no lubricant<br>- ( ) Other /Specify_______________<br><br>0- ( ) No response	
**P1211**	The last time you used a condom with a client; did you use the condom all the time for penetration?	1- ( ) Yes<br>2- ( ) No<br><br>0- ( ) No response	
**P1212**	The last time you had sex with a client; did you use lubricant during intercourse?	1- ( ) Yes<br>2- ( ) No<br><br>0- ( ) No response	
**P1213**	In general, how often do you use a condom during sex with your clients?	1- ( ) All the time<br>2- ( ) Most of the times<br>3- ( ) Often<br>4- ( ) Few times<br>5- ( ) Almost never<br>6- ( ) Never<br><br>0- ( ) No response	
**P1214**	In general, how often do you use lubricant during sex with your clients?	1- ( ) All the time<br>2- ( ) Most of the times<br>3- ( ) Often<br>4- ( ) Few times<br>5- ( ) Almost Never<br>6- ( ) Never<br><br>0- ( ) No response	
**P1215**	You stop using a condom with a client when...?<br><br>**PROBE FOR EACH PROBLEM AND CHECK THOSE THAT PERSON HAS HAD**	- ( ) Uses drugs or alcohol<br>- ( ) Is a f requent client<br>- ( ) The client pays more<br>- ( ) The client insists<br>- ( ) Does oral sex<br>- ( ) Checks the client and looks healthy<br>- ( ) Other /Specify_______________<br><br>0- ( ) No response	

SECTION 13: DEMOGRAPHIC CHARACTERISTICS

No.	Questions	Categories	Go to
**P1301**	What is your date of birth?	____Day_____Month__________year<br><br>00000000- ( ) Does not know	
**P1302**	How old are you?	Age __________<br><br>0- ( ) No response	
**P1303**	If by gender identity it is understood or subjective perception that a person has about himself / herself as a man or woman, how would you define your gender identity?<br><br>**READ THE OPTIONS**	1- ( ) Female<br>2- ( ) Male<br>3- ( ) Female trans<br>4- ( ) Male trans<br>5- ( ) Other /Specify ________________<br><br>0- ( ) No response	
**P1304**	Do you know how to read and write?	1- ( ) Yes<br>2- ( ) No..<br><br>0- ( ) No response................................	→ **P1306**
**P1305**	What level of education do you have?	1- ( ) Did not go to school<br>2- ( ) Literate<br>3- ( ) Incomplete primary school<br>4- ( ) Complete primary school<br>5- ( ) Incomplete High School<br>6- ( ) Complete High School<br>7- ( ) Incomplete University<br>8- ( ) Complete University<br>9- ( ) Post college degree<br><br>0- ( ) No response	
**P1306**	What is your Religion?	1- ( ) Catholic<br>2- ( ) Evangelical Christian<br>3- ( ) Jehovah Witness<br>4- ( ) Adventist<br>5- ( ) Mormon<br>6- ( ) Other /Specify________________<br><br>7 ( ) None……………………………………..<br>0- ( ) No response…………………………..	→ **P1308**
**P1307**	How many times per month do you go to church?	1 ( ) At least once a week<br>2 ( ) One, two or three times per month<br>3 ( ) Less than once a month<br>4 ( ) Never<br><br>0- ( ) No response	

P1308	Do you live permanently in this country?	1- ( ) Yes<br>2- ( ) No<br><br>0- ( ) No response	
**No.**	**Questions**	**Categories**	**Go to**
P1309	From the moment you learned you had HIV, have you changed your place of residence?	1- ( ) Yes…………………………………………<br>2- ( ) No<br><br>0- ( ) No response………………………………	→ P1312<br><br><br>→ P1312
P1310	What was the main reason why you changed residence?	1- ( ) To search for a job<br>2- ( ) For work purposes<br>3- ( ) Persecution or discrimination for being a person with HIV<br>4- ( ) Other /Specify ________________<br><br>0- ( ) No response	
P1311	Did you have any of these problems to relocate?<br><br>**PROBE FOR EACH PROBLEM AND CHECK THOSE THAT APPLY**	- ( ) Access to antiretroviral treatment<br>- ( ) Access condoms<br>- ( ) Access lubricants<br>- ( ) Access to health services<br><br>0- ( ) No response	
P1312	What is your present marital status?	1- ( ) Single<br>2- ( ) Married / common law 3-<br>( ) Separated or divorced 4- (<br>) Widower<br>0- ( ) No response	
P1313	At the moment with whom do you live with?<br><br>**YOU MAY MARK MORE THAN ONE OPTION**	- ( ) Lives with partner<br>- ( ) Lives with the family of the partner<br>- ( ) Lives with his / her family<br>- ( ) Lives alone<br>- ( ) Lives with a f riend<br>- ( ) Other / Specify ________________<br>0- ( ) No response	
P1314	What do you do?	1- ( ) Only works<br>2- ( ) Studies and works<br>3- ( ) Unemployed …………………….<br>4- ( ) Has a pension ……………..<br>5- ( ) Only Studies……………………………<br>6- ( ) Is devoted to housework…………….<br>7- ( ) Does not study or work and does do housework……………………………<br><br>0- ( ) No response……………………………	→ **FIN DE ENTRE-VISTA**
P1315	What is your main occupation and what post do you have?	________________________________	

P1316	What are the main activities or tasks you perform at work?	______________________________________	
**No.**	**Questions**	**Categories**	**Go to**
**P1317**	At work you are……. <br>**READ OPTIONS**	1- ( ) Unpaid 2- ( ) Government or Institution employee 3- ( ) Employee of a private company 4- ( ) Domestic worker 5- ( ) Self employed 6- ( ) Employer with fewer than 10 employees 7- ( ) Employer with more than 10 employees 0- ( ) No response	
**P1318**	In addition to the employment already mentioned, do you have another source of income?	1- ( ) Yes 2- ( ) No <br>0- ( ) No response	
**P1319**	Could you tell us what your income is or how much you earn monthly? <br>**MAKE SURE THAT THE SOURCE IS PER MONTH AND NOT WEEKLY** <br>**IF PERSON INDICATES ANOTHER CURRENCY THAT IS NOT THAT OF THE COUNTRY MAKE THE CALCULATION TO COUNTRY CURRENCY**	Amount per month :____________ <br>Currency: __________ <br>01- ( ) Does not know <br>00- ( ) No response	
**P1320**	How many people depend on you f inancially? <br>**THIS IINCLUDES CHILDREN, PARENTS, FAMILY MEMEBERS AND OTHER PEOPLE.**	Number of people: __________ <br>99- ( ) No response	

THANK YOU FOR YOUR TIME AND THE INFORMATION PROVIDED!
